# RecQ-core of BLM unfolds telomeric G-quadruplex in the absence of ATP

**DOI:** 10.1093/nar/gku856

**Published:** 2014-09-22

**Authors:** Jagat B. Budhathoki, Sujay Ray, Vaclav Urban, Pavel Janscak, Jaya G. Yodh, Hamza Balci

**Affiliations:** 1 Department of Physics, Kent State University, Kent, OH 44242, USA; 2 Institute of Molecular Genetics AS CR, Prague, Czech Republic; 3 Institute of Molecular Cancer Research, University of Zurich, Zurich, Switzerland; 4 Department of Physics and Center for the Physics of Living Cells, University of Illinois at Urbana-Champaign, Urbana, IL 61801, USA

## Abstract

Various helicases and single-stranded DNA (ssDNA) binding proteins are known to destabilize G-quadruplex (GQ) structures, which otherwise result in genomic instability. Bulk biochemical studies have shown that Bloom helicase (BLM) unfolds both intermolecular and intramolecular GQ in the presence of ATP. Using single molecule FRET, we show that binding of RecQ-core of BLM (will be referred to as BLM) to ssDNA in the vicinity of an intramolecular GQ leads to destabilization and unfolding of the GQ in the absence of ATP. We show that the efficiency of BLM-mediated GQ unfolding correlates with the binding stability of BLM to ssDNA overhang, as modulated by the nucleotide state, ionic conditions, overhang length and overhang directionality. In particular, we observed enhanced GQ unfolding by BLM in the presence of non-hydrolysable ATP analogs, which has implications for the underlying mechanism. We also show that increasing GQ stability, via shorter loops or higher ionic strength, reduces BLM-mediated GQ unfolding. Finally, we show that while WRN has similar activity as BLM, RecQ and RECQ5 helicases do not unfold GQ in the absence of ATP at physiological ionic strength. In summary, our study points to a novel and potentially very common mechanism of GQ destabilization mediated by proteins binding to the vicinity of these structures.

## INTRODUCTION

Human Bloom helicase (BLM) is a member of RecQ family (1,2), which includes *Escherichia coli* RecQ, *Saccharomyces cerevisiae* Sgs1p, *Schizosaccharomyces pombe* Rqh1 and human WRN, RECQL1, RECQL4 and RECQL5 helicases (3–7). RecQ helicases share a central domain that includes Walker A and B box which bind and hydrolyze adenosine triphosphate (ATP). Deficiencies in BLM cause Bloom syndrome, which is marked by genomic instability and increased predisposition to cancer, infertility and dwarfism (8,9). BLM is particularly important in suppressing reciprocal exchanges between sister chromatids. BLM has been shown to form a hexameric ring (10) and other multimeric structures (11). However, BLM mutants lacking the oligomerization domain can unwind dsDNA, in the 3′ to 5′ direction (12). BLM works as part of a larger multiprotein complex called the ‘dissolvasome’ or BTR complex (a complex of BLM, topoisomerase IIIα, RMI1 and RMI2) to remove non-canonical DNA structures as part of homologous recombination-dependent repair during DNA replication and telomere maintenance (13,14). Even though BLM is capable of forming multimeric structures, recent studies suggest that such structures dissociate upon ATP hydrolysis and it is the BLM monomer that unwinds dsDNA and resolves non-canonical DNA structures, such as Holliday junctions and D-loops (11).

G-quadruplex structures (GQs) are non-canonical DNA structures that form in guanine-rich regions of the genome. GQs are stabilized by Hoogsteen hydrogen bonding between guanosines, stacking of G-tetrad layers, coordination of Hoogsteen bonds by monovalent cations that intercalate between the tetrad layers and by hydration (15–18). Telomeric DNA is capable of folding into GQs in eukaryotes (19–21) and GQs are considered to take part in maintenance and elongation of telomeres (22,23). In addition, potentially intramolecular GQ forming sequences (PQS) have been computationally identified in several hundred thousand sites in the human genome (19,24–26). PQS are particularly concentrated in promoter sites (27–31), suggesting a potential role in transcription level gene expression regulation (32,33). Therefore, GQs have been targeted by specific drugs to modulate gene expression (34). Similarly, RNA GQs in the 5′-UTR region (30) have been demonstrated to regulate translation level gene expression (35–38). GQs were recently shown to exist in human cells being modulated during the cell cycle (39), further highlighting the physiological relevance of studying the interactions between these structures and relevant proteins. GQs are typically thermally very stable and require protein activity to be unfolded (40–43). Genome-wide studies have shown that eliminating helicases that have GQ unfolding activity, such as *S. cerevisiae* Pif1 or human BLM, resulted in increased DNA breaks in PQS and severe retardation of DNA replication (41,44–48). It was recently shown that Pif1 suppresses genome instability at GQ motifs (48). In addition, in Sgs1 deficient cells, mRNA levels from regions of the genome rich in PQS were repressed at significantly higher levels relative to other regions, including G-rich regions unable to form GQ (49). These measurements suggested that Sgs1 targets GQ and destabilizes it, enabling transcription machinery to proceed. Similar results were obtained in BLM deficient human cells as well (47). In addition, BLM was proposed to have a genome-wide activity of resolving regions that are difficult to replicate, including telomeres, which could form GQs (13).

*In vitro* studies have demonstrated that both Sgs1 and BLM bind GQ with high affinity (*K_D_* ≈5 nM) and can efficiently unfold intermolecular GQs (50–53). Electron microscopy studies showed that BLM preferentially interacts with GQ located within a much longer dsDNA, which is a better representative of genomic DNA compared to isolated short GQ constructs (54). A consensus exists among various studies on the requirement of a 3′ overhang for successful unfolding of GQ by BLM (55). However, the majority of the previous studies probing BLM-GQ interactions were performed on intermolecular GQs. Interactions with intramolecular GQs, which are more likely to form in the PQS regions of the genome, were only recently studied using bulk fluorescence quenching and polarization methods (56). This study concluded that BLM unfolds intramolecular GQs with much lower efficiency compared to unwinding dsDNA, which is in contrast to the intermolecular GQ case. Other conclusions of this study were that BLM unfolding of intramolecular GQ requires ATP and is inversely proportional to the stability of the GQ structure.

In this study, we present findings that challenge the current understanding of the mechanism of BLM-mediated GQ unfolding, and helicase-GQ interactions in general. We used single molecule Förster Resonance Energy Transfer (smFRET) to measure unfolding of intramolecular GQ by RecQ-core of BLM in the nucleotide-free (nt-free), ATPγS and ADP states under physiological salt concentrations. This truncated BLM construct, lacking the oligomerization region, was used to study the activity of BLM monomers (12) and will simply be referred to as BLM in the rest of the manuscript. We used DNA constructs of human telomeric repeat that form a single intramolecular GQ and terminate with a 3′ overhang of 2–15 nt. We have found that BLM-mediated GQ unfolding activity is most efficient in the ATPγS-bound state (will be referred to as ATPγS state), followed by the nt-free state and then the ADP-bound state (will be referred to as ADP state). This order correlates with the binding stability of BLM to the overhang ssDNA for these nucleotide states. In addition, we observed BLM-mediated GQ unfolding for overhang lengths as short as 6 nt, but not for shorter overhangs. Enhancing GQ stability by shortening the loops resulted in a decrease in BLM-mediated GQ unfolding, and reducing GQ stability by using lower ion concentration increased GQ unfolding. We observed significantly less BLM-mediated GQ unfolding with a 5′ overhang compared to a 3′ overhang. Finally, RecQ and RECQ5 showed much weaker GQ unfolding activity compared to BLM. In contrast, WRN had a similar GQ unfolding activity as BLM.

## MATERIALS AND METHODS

### DNA and proteins

**Figure 1.**
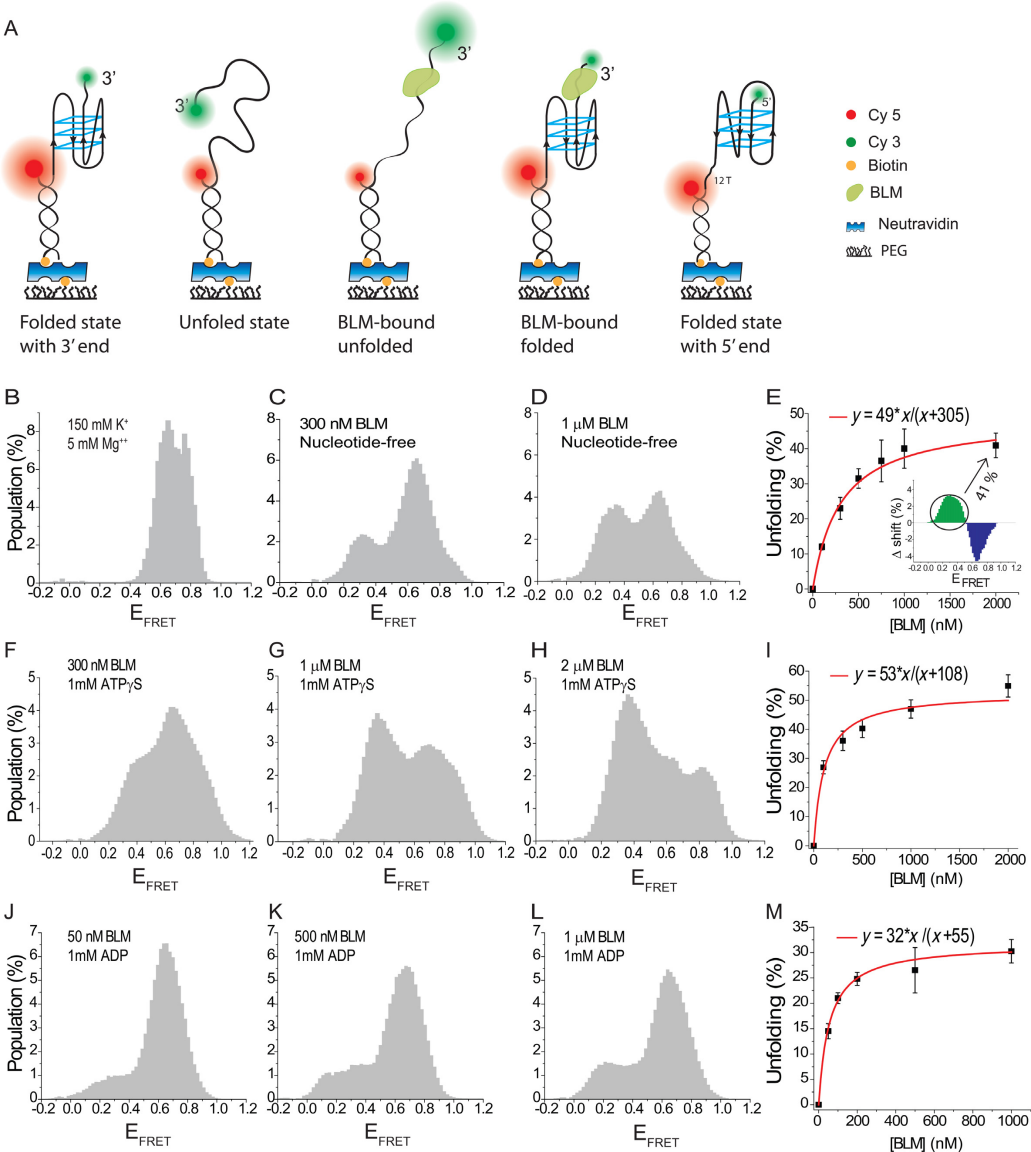
BLM-mediated unfolding for pd-hGQ12T (the GQ construct with 12 nt long 3′ overhang) in which BLM concentration is titrated in nt-free, 1 mM ATPγS, or 1 mM ADP states. All measurements were performed at 150 mM K^+^. (A) Cartoons depicting different DNA and BLM-DNA complex conformations that are referred to in the manuscript. The size of circles representing the donor (green) and acceptor (red) fluorophores are drawn to reflect their relative brightness in the relevant conformation. The folded GQ state has the highest E_FRET_ and the BLM-bound unfolded state has the lowest E_FRET_. (B)–(E) BLM titration in the nt-free state. (B) The folded GQ state before BLM is introduced. (C) Adding 300 nM BLM results in emergence of a low FRET peak. (D) Adding 1 μM BLM further increases the low FRET peak population. (E) BLM-mediated GQ unfolding is quantified by subtracting the FRET distribution of folded GQ, shown in (B), from the FRET distribution at various BLM concentrations. The inset shows a representative histogram that results from this subtraction. The cumulative positive area is plotted as a function of BLM concentration. The redline is a Langmuir isotherm fit to the data. (F)–(I) BLM-mediated GQ unfolding at 1 mM ATPγS for different BLM concentrations. (F) 300 nM BLM; (G) 1 μM BLM; (H) 2 μM BLM. (I) Similar analysis as in (D) is performed for the ATPγS data. The redline is a Langmuir isotherm fit to the data. (J)–(M) BLM-mediated GQ unfolding at 1 mM ADP for different BLM concentrations. (J) 100 nM BLM; (K) 500 nM BLM; (L) 1 μM BLM. (M) A summary of the data at 1 mM ADP and a Langmuir binding isotherm fit to these data.

The GQ forming DNA constructs used in our study, with one exception, contained the human telomeric sequence, (GGGTTA)_3_GGG, as well as a 3′ overhang of 2–15 nt. For the exception, we used a sequence that forms a three layer GQ with 1 nt loops: (GGGT)_3_GGG. The sequences of these constructs (purchased from and high pressure liquid chromatography purified by Integrated DNA Technologies, Coralville, IA, USA) are given in Table 1. The underlined segment of sequences in Table 1 folds into GQ upon addition of K^+^ ions. In order to minimize the interaction of the GQ and BLM with the surface, partial duplex DNA (pdDNA) constructs with an 18 base pair long RNA/DNA heteroduplex stem and a flanking 3′ ssDNA were used. The flanking 3′-ssDNA contains the GQ forming segment and the overhang of 2, 4, 6, 8, 10, 12 or 15 nt (hGQxT or hGQ4nt in Table 1). With the exception of 4 nt overhang, which has a TTAG sequence, all other overhangs are polythymine. The pdDNA constructs were formed by annealing the GQ containing ssDNA with the RNA-Stem (Table 1), and are called pd-hGQxT with *x* = 2, 6, 8, 10, 12 or 15. The construct with 4 nt overhang is called pd-hGQ4nt. In pdDNA constructs, Cy5 and biotin are on the RNA-Stem and Cy3 is at the flanking 3′-end. The exception to this is pd-hGQ12T-5′, the construct with a free 5′ end, which has a Cy5 at the 5′-end and a Cy3 and biotin on the DNA-Stem. See Figure 1A for schematics of the constructs. In addition to these GQ forming constructs, three DNA constructs that did not form GQ or any other secondary structure, polyT15, polyT15–5′ and polyT35, were annealed with the RNA-Stem to form pd-polyT15, pd-polyT15–5′ and pd-polyT35, respectively. These constructs were used for measurements in which binding of BLM to the ssDNA in the absence of GQ were probed.

**Table 1.** The DNA constructs used in these studies

Name	Sequence
**hGQxT**	5′- TGG CGA CGG CAG CGA GGC TAG GGT TAG GGT TAG GGT TAG GG (xT) Cy3
	*x* = 2, 6, 8, 10, 12, or 15
**hGQ4nt**	5′- TGG CGA CGG CAG CGA GGC TAG GGT TAG GGT TAG GGT TAG GG TTAG Cy3
**3Ly1Lp12T**	5′- TGG CGA CGG CAG CGA GGC TTG GGT GGG TGG GTG GG(12T) Cy3
**hGQ12T-5′**	5′-Cy5-(12T) GGG TTA GGG TTA GGG TTA GGG TT TGG CGA CGG CAG CGA GGC
**12ThGQ-5′**	5′-Cy3-TT GGG TTA GGG TTA GGG TTA GGG(12T) TGG CGA CGG CAG CGA GGC
**polyT15**	5′- TGG CGA CGG CAG CGA GGC (15T) Cy3
polyT15–5′	5′-Cy5-(15T) TGG CGA CGG CAG CGA GGC
**polyT35**	5′-TGG CGA CGG CAG CGA GGC (35T) Cy3
RNA-Stem	5′- Cy5 GCC UCG CUG CCG UCG CCA Biotin
DNA-Stem	5′- Biotin GCC TCG CTG CCG TCG CCA Cy3

The constructs whose names are in bold letters are hybridized with the RNA-Stem to form a pdDNA. polyT15–5′ and DNA-Stem are hybridized to form pd-polyT15–5′. The underlined nucleotides form the GQ structure. The number of consecutive thymines are written in parenthesis, e.g. (12T) means 12 consecutive thymines.

A truncated core BLM (BLM^642–1290^) was purified and characterized as described (57). In addition, mutant form of core BLM, BLM^K695M^, carrying a K695M substitution in helicase motif I was generated using QuikChange Site-Directed Mutagenesis Kit (Stratagene), and purified using a similar protocol to core BLM. Core BLM lacks the oligomerization domain, and therefore all experiments in this report are performed with monomeric BLM. BLM protein was stored in storage buffer that contains 50 mM Tris-HCl (pH = 7.5), 200 mM NaCl, 50% (v/v) glycerol and 1 mM dithiothreitol (DTT), and diluted in imaging solution, described in the smFRET Assay section below, to the desired concentration before being added to the microfluidic channel. Purification of *E. coli* RecQ (58), human full length RECQ5 (59) and full length WRN (60) helicases was performed according to protocols in the cited references.

### Sample preparation

To create the sample chamber, double-sided tape was sandwiched between a quartz slide and a glass cover slip. The quartz slide was first drilled, cleaned and coated with polyethylene-glycol (PEG) and biotin-PEG in the ratio of ∼100:1 (m-PEG-5000:biotin-PEG-5000 from Laysan Bio Inc.). Nucleic acid immobilization on the surface was achieved via a neutravidin-biotin linker. DNA and RNA-stems were hybridized by annealing them at 95°C for 5 min., followed by cooling to room temperature over 2–3 h. A stock concentration of 1 μM was prepared for this annealing step, which was diluted to 15 pM in multiple steps before injection into chamber. After 1–2 min of incubation, the chamber was washed to remove excess DNA. A density of roughly 250 molecules/imaging area (∼5 × 10^3^ μm^2^) is obtained as a result of this protocol. Note that all experiments were carried out with samples that had undergone annealing the same day.

### smFRET assay

A prism-type total internal reflection microscope built around an Olympus IX-71 microscope was used for all the measurements. Data were acquired at 40 ms time resolution using Andor Ixon EMCCD camera (iXon DV 887-BI EMCCD, Andor Technology, CT, USA). The imaging solution used in all measurements contained Tris base (50 mM, pH 7.5), 2 mM trolox, 0.8 mg/ml glucose, 0.1 mg/ml bovine serum albumin, 1 mM DTT, 0.1 mg/ml glucose oxidase, 0.02 mg/ml catalase, 5 mM MgCl_2_ and 150 or 50 mM KCl. The few cases in which NaCl was used instead of KCl, to reduce the stability of GQ structure, are explicitly stated. As the cation concentration (K^+^) is significant for GQ stability, we henceforth refer to KCl as K^+^. In all assays performed in this study, the Mg^++^ was kept at 5 mM, regardless of monovalent cation type or concentration. Therefore, only the cation concentration will be mentioned where relevant, but it should be assumed that 5 mM Mg^++^ is also present in all assays. BLM as well as ATP, ADP and ATPγS were mixed in imaging solution at the desired concentration. The imaging solution was incubated with the DNA sample for 15 min to allow the system to reach steady state. Longer incubation times up to 1 h did not have a significant effect on the smFRET distributions. Long (1000–4000 frames) and short movies (30 frames) were recorded for two different types of analysis.

### Data analysis and quantification of BLM-mediated GQ unfolding

**Figure 2.**
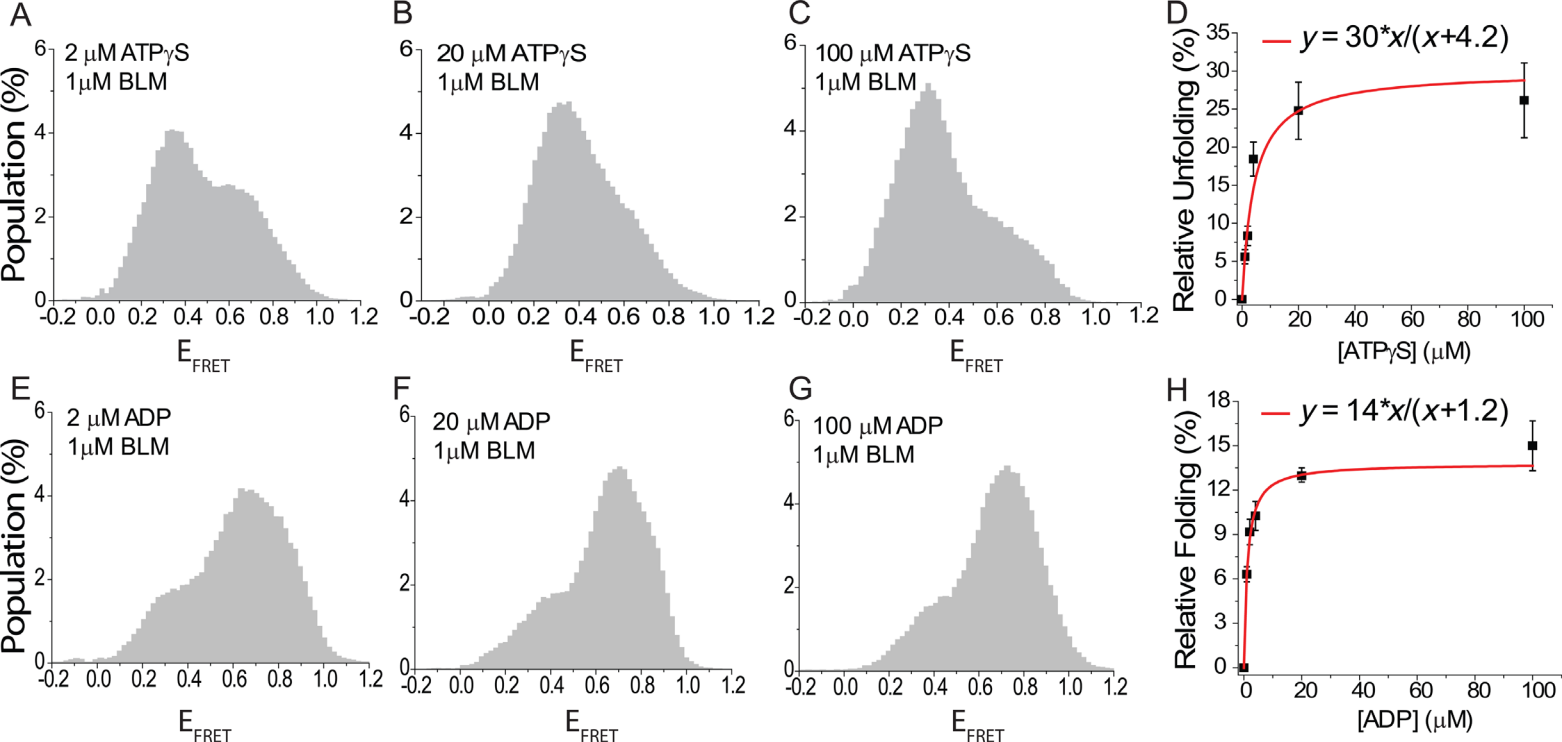
BLM-mediated unfolding for pd-hGQ12T in which ATPγS or ADP is titrated while BLM concentration is kept at 1 μM (150 mM K^+^, pH 7.5). (A)–(D) ATPγS titration data. (A) 2 μM ATPγS; (B) 20 μM ATPγS; (C) 100 μM ATPγS. (D) Subtraction analysis as in Figure 1E. The histogram for 1 μM BLM data, before any ATPγS is introduced as shown in Figure 1D, is subtracted from the histograms for different ATPγS concentrations in the presence of 1 μM BLM. This choice ensures that the change in the unfolded population is due to the changing ATPγS concentration. As the reference state already shows some GQ unfolding, the *y*-axis of the graph represents relative unfolding with respect to this reference state. The redline is a Langmuir binding isotherm fit to the data. (E)–(H) ADP titration data. The low E_FRET_ peak decreases and the high E_FRET_ peak increases as the ADP concentration is increased, representing an increase in the folded GQ population. (E) 2 μM ADP. (F) 20 μM ADP. (G) 100 μM ADP. (H) Subtraction analysis in which the reference histogram is that for 1 μM BLM in the absence of ADP (nt-free state with 1 μM BLM shown in Figure 1D). Unlike the previous cases, the folded population increases as ADP concentration is increased, which shows that BLM is less efficient at unfolding GQ in the ADP state compared to the nt-free state. The relative folded population is thus plotted in (H) to maintain a positive population.

Long movies were analyzed using custom software to generate intensity and FRET efficiency (E_FRET_) time traces for each molecule. Such traces were filtered to ensure that only single molecules were selected and the background was subtracted from each of these selected molecules using a custom Matlab code. These traces were used to build population histograms of E_FRET_. Folded GQ constructs show high E_FRET_ ≈ 0.60–0.80, whereas unfolded and protein-bound unfolded constructs show significantly lower E_FRET_, which depend on the construct used but is typically <0.40. The histograms were normalized to a percentage scale such that the total number of molecules in the histogram represents 100%. This normalization is necessary for the subtraction analysis which is employed to quantify the effect of BLM or nucleotides (effectors) on the E_FRET_ distribution. In this analysis, a normalized reference histogram, i.e. the histogram representing the state that does not contain the titrated effector, is subtracted from normalized histograms representing various effector concentrations. The relevant reference histogram used for each subtraction analysis is specifically mentioned in the related section describing the data. In general, if BLM is titrated (at a constant nucleotide concentration), the histogram at zero BLM concentration is used as the reference. On the other hand, if a nucleotide is titrated (at constant BLM concentration) then the histogram at zero nucleotide concentration (in the presence of relevant BLM concentration) is used as the reference. For example, for BLM titration experiments in Figure 1B–E, the reference histogram is the folded GQ histogram (at 150 mM K^+^) before BLM is introduced to the chamber (Figure 1B). This reference is subtracted from the normalized histograms of BLM-titrations (Figure 1C and D in this case). On the other hand, to quantify the influence of 20 μM ATPγS on BLM-mediated GQ unfolding, the FRET distribution at 1 μM BLM and zero ATPγS concentration (Figure 1D) was subtracted from the distribution at 1 μM BLM and 20 μM ATPγS (Figure 2B). The subtraction results in a distribution with equal positive and negative populations since both the minuend and subtrahend histograms are normalized to 100% (see inset of Figure 1E). The negative population at the higher E_FRET_ represents the decrease in the folded GQ population, which results upon adding BLM in a particular nucleotide concentration to the chamber. The total positive population at the lower E_FRET_ region represents the unfolded and BLM-bound unfolded GQ population. This analysis method was preferred over fitting multiple Gaussian peaks to smFRET distributions and quantifying the population of each peak as a function of effector concentration. The main reason behind this choice was the difficulty in uniquely identifying the peaks that would best fit the relatively broad FRET distributions.

Finally, Langmuir binding isotherm fit, of the form *y* = *αx*/(*x* + *K*_eq_), is used to analyze the results obtained from these subtractions. The independent variable *x* is the effector concentration (either BLM or nucleotide concentration depending on the measurement), and the dependent variable *y* is the percent unfolded population with respect to a reference state. In this expression, *K*_eq_ is the equilibrium constant and *α* describes the maximum unfolded population under saturating effector concentration. The error bars associated with the data were obtained from standard deviations of multiple data sets obtained for a given condition. The error values for the parameters of the Langmuir binding isotherm fits were obtained as a result of a weighted fit, i.e. the uncertainties in the individual data points were taken into account while fitting the data. Subtraction analysis and fits to data were performed using Origin Pro 8.5.

### Identification of the observed FRET levels

The coiled DNA is stabilized into a GQ structure by K^+^ ions, resulting in a high E_FRET_. Interaction of BLM with these folded GQ constructs results in various states with lower E_FRET_ (Figure 1A). The observed states from highest to lowest E_FRET_ are: folded GQ, BLM-bound folded GQ (i.e. BLM bound to the overhang with GQ remaining folded), unfolded GQ (or unfolded DNA which is essentially coiled DNA that is not bound by BLM) and BLM-bound unfolded DNA. E_FRET_ for the folded GQ is determined in the presence of 150 mM K^+^, the physiological concentration, before BLM is added to the sample chamber. In a few cases either 50 mM K^+^ or 50 mM Na^+^ was used instead, and is explicitly stated. In the case of pd-hGQ12T, which is a pdDNA GQ construct with a 12 thymine long overhang formed by hybridizing hGQ12T and RNA-Stem of Table 1, the folded state shows a broad distribution that can be fit by two Gaussian functions with E_FRET_ = 0.64 ± 0.07 and E_FRET_ = 0.78 ± 0.05. In human telomeric constructs with shorter overhangs, these multiple peaks were interpreted as signatures of different folding conformations, which are also observed in circular dichroism (CD) and NMR studies (61). We sought to determine the FRET efficiencies of unfolded and BLM-bound unfolded states of pd-hGQ12T by using a pdDNA construct with a polythymine tail of similar length as a reference. This approach avoids the complications that might arise due to possible secondary structure formation of pd-hGQ12T. The ssDNA part of pd-hGQ12T (GQ forming segment which is 21 nt plus an additional 2 nt on the 5′ side and a 12 nt 3′-overhang) is 35 nt long which is the same length as that of pd-polyT35. Therefore, the two constructs would result in very similar FRET efficiencies in the unfolded (coiled) and BLM-bound unfolded states. This analysis results in E_FRET_ = 0.40 ± 0.06 for the unfolded state, and E_FRET_ = 0.20 ± 0.08 for the BLM-bound unfolded state (Supplementary Figure S1). The BLM-bound folded state would have an E_FRET_ between the folded and unfolded states, hence E_FRET_ ≈ 0.55, which is distinctly observed in single molecule traces (data not shown) and is also visible in the steady-state histograms in Figures 1 and 2. E_FRET_ for the unfolded states should be similar for the two main constructs of this study, pd-hGQ12T and pd-hGQ15T, as they only differ in length by 3 nt. Correspondingly, E_FRET_ for their BLM-bound unfolded states should be similar. Therefore, the E_FRET_ levels measured for pd-polyT35 for unfolded and BLM-bound unfolded states should also be similar to those of pd-hGQ15T, respectively. pd-polyT12 and pd-polyT12–5′ differ only in terms of the directionality of their overhangs but otherwise have the same separation between their donor–acceptor pairs. Therefore, the E_FRET_ values for these constructs should be the same.

### Electrophoretic mobility shift assay (EMSA)

BLM binding to pd-hGQ12T construct was measured by EMSA. Native polyacrylamide gel electrophoresis (PAGE) was used for this measurement. The partial duplex construct was formed by annealing the two strands via heating/slow cooling prior to the gel binding assay. Annealed substrates were mixed with varying BLM concentrations (0–500 nM) in 50 mM K^+^, 5 mM Mg^++^, 50 mM Tris-HCl (pH 7.5) and 1 mM DTT. Binding reactions were carried out for 10 min at 22°C and then mixtures were loaded onto a 4–20% polyacrylamide TBE gel and run in 0.5× TBE at 50V for 5 h at 4°C. Gel was imaged using a Molecular Dynamics Typhoon 9400 Multilaser Scanner.

## RESULTS

### ATP binding or hydrolysis is not required for BLM-mediated intramolecular GQ unfolding

We employed smFRET assay to measure intramolecular GQ unfolding activity of BLM in nucleotide-free (nt-free), ATPγS and ADP states under physiological salt concentrations (150 mM K^+^). In this study we used a core BLM polypeptide, BLM^(642–1290)^, that exists only as a monomer both in solution and in the DNA-bound state (57). Figure 1B shows the folded GQ state before BLM is introduced to the chamber for pd-hGQ12T (see Figure 1A for a schematic). The smFRET distribution representing the folded state did not change when 1 mM ATPγS or 1 mM ADP were added to the chamber in the absence of BLM (data not shown). Therefore, the folded state in Figure 1B is used as the reference state for the subtraction analysis for all nucleotide states presented in Figure 1. Figure 1C shows the smFRET distribution when 300 nM BLM is added to the chamber in the nt-free state. A low FRET population, at E_FRET_ ≈ 0.30, representing BLM-mediated GQ unfolding accumulates as the BLM concentration is increased to 1 μM BLM (Figure 1D). As this FRET level is in between unfolded (E_FRET_ = 0.40 ± 0.06) and BLM-bound unfolded (E_FRET_ = 0.20 ± 0.08) states identified in Supplementary Figure S1, an equilibrium of both states is represented by the peak at E_FRET_ = 0.30. Figure 1E shows a summary of the subtraction analysis performed for different BLM concentrations. The reference data for the subtraction analysis were the folded state in Figure 1B. Langmuir binding isotherm fit (red curve in Figure 1E) to the data yields an equilibrium constant of *K*_eq_ = 305 ± 16 nM and *α* = 49 ± 1%, (i.e. at saturating BLM concentration 49% of all initially folded GQ molecules are unfolded). We have further confirmed BLM-mediated GQ unfolding in the absence of ATP with a CD assay. In this assay, we measured the CD spectrum of DNA before and after adding 1 μM BLM to the environment. The characteristic peaks indicating GQ formation are diminished after adding BLM to the environment, indicating destabilization of GQ (Supplementary Figure S2A). Finally, smFRET measurements were performed on a DNA construct in which donor–acceptor fluorophores are moved directly to the ends of the GQ. This construct eliminates any significant FRET changes that might take place due to binding of BLM to the overhang, and results in lower FRET efficiency only when GQ is unfolded. The schematic of this construct and the smFRET data are shown in Supplementary Figure S2B. Even though this construct resulted in a reduction in the amount of unfolding in 150 mM K^+^, probably due to the internal fluorophore interfering with BLM-GQ interactions, a significant low FRET population, representing unfolded GQ was observed in 50 mM and 10 mM K^+^ (Supplementary Figure S2C and D). As is clear from these different measurements, BLM does not need ATP or any other nucleotide to unfold an intramolecular GQ at physiological pH and ionic strength. This is a novel observation as earlier studies performed on intermolecular GQs (50,52), as well as a previous bulk study on an intramolecular GQ (56), reported BLM-mediated unfolding only in the presence of ATP.

Figure 1F–I show BLM titration experiments in the presence of 1 mM ATPγS. The folded GQ state before BLM is introduced is not shown as it is identical to that in Figure 1B. Similar to the nt-free state, a low FRET population emerges and increases as BLM concentration is increased. However, this low FRET population is clearly greater for any given BLM concentration in the ATPγS state compared to the nt-free state. This is also reflected in the Langmuir binding isotherm fit in Figure 1I which yields an equilibrium constant of *K*_eq_ = 108 ± 24 nM, 3-fold lower than that of the nt-free state, and *α* = 53 ± 3%, which is same as that of the nt-free state within the uncertainties of the measurements. The effect of ATPγS on GQ-unfolding by BLM is substrate-specific as we have previously shown that BLM could not unwind traditional forked DNA substrates in the presence of ATPγS (12). This result was confirmed under our assay conditions as well.

Figure 1J–M show BLM titration measurements in the presence of 1 mM ADP. BLM-mediated GQ unfolding is less efficient in the ADP state compared to the nt-free and ATPγS states. Langmuir binding isotherm fit in Figure 1M results in *K*_eq_ = 55 ± 5 nM and *α* = 32 ± 1%. The lower *α* parameter suggests that in steady state, a lower fraction of the GQ molecules are unfolded by BLM in saturating ADP concentration compared to the nt-free and ATPγS states. Fitting parameters obtained from all Langmuir isotherm fits performed in the manuscript are summarized in Table 2. The smFRET histograms and the subtraction analysis for BLM concentrations that are not shown in the manuscript are presented in Supplementary Figures S3 and S4.

**Table 2.** A summary of Langmuir binding analysis performed for pd-hGQ12T

Summary of Langmuir Isotherm fits *y* = *αx*/(*x* + *K*_eq_)						
State	K^+^ Con.	Effector	Reference state	*α* (%)	*K*_eq_	Figure
Nt-free	150 mM	BLM	Folded state	49 ± 1	305 ± 16 nM BLM	Figure 1E
1 mM ATPγS	150 mM	BLM	Folded state	53 ± 3	108 ± 24 nM BLM	Figure 1I
1 mM ADP	150 mM	BLM	Folded state	32 ± 1	55 ± 5 nM BLM	Figure 1M
1 μM BLM	150 mM	ATPγS	1 μM BLM, Nt-free	30 ± 4	4.2 ± 1.1 μM ATPγS	Figure 2D
1 μM BLM	150 mM	ADP	1 μM BLM, Nt-free	14 ± 1	1.2 ± 0.1 μM ADP	Figure 2H
1 μM BLM	150 mM	AMP-PNP	1 μM BLM, Nt-free	18 ± 1	2.1 ± 0.3 AMP-PNP	Figure S11

All assays include 5 mM Mg^++^ in addition to the indicated K^+^ concentration. Folded state refers to the state in the presence of the indicated K^+^ concentration but in the absence of BLM. In the titrations where ATPγS or ADP is the effector, the reference state is selected as the nt-free state in the presence of BLM (at the indicated concentration) to reflect the influence of nucleotide on BLM-mediated GQ unfolding. Note that isotherm fit parameters cannot be compared if the reference state is different. Also, note that the fit parameters for ADP titration at 1 μM BLM, last row of table, represent relative folding rather than unfolding.

We performed several control and complementary measurements to study BLM-mediated GQ unfolding in the absence of ATP and eliminate alternative explanations. We provided an independent determination of BLM binding to GQ-forming construct using a native PAGE EMSA with pd-hGQ12T substrate. A single shifted band was observed as BLM was titrated, although the GQ folding state upon BLM binding is not discernible in this assay (Supplementary Figure S5). No GQ unfolding was observed upon addition of storage buffer without BLM or in the presence of heat-denatured BLM, confirming that activity required native BLM and was not due to GQ-destabilizing elements, such as metal chelators in the buffer (Supplementary Figure S6). We ruled out the possibility of ATP contamination in purified BLM by testing whether BLM could unwind a forked DNA substrate (12) in the absence of any additional ATP. No unwinding of this substrate was observed in the nt-free or ATPγS states but activity was measured only upon addition of ATP (Supplementary Figure S7). We eliminated the possibility that the GQ unfolding activity we observe may be due to another protein co-purifying with BLM that possesses GQ unfolding activity in the absence of ATP (e.g. a ssDNA binding protein), as sodium dodecyl sulphate-PAGE analysis of our purified BLM displayed a single band that corresponds to the expected molecular weight of BLM (Supplementary Figure S8).

Finally, to ensure BLM-mediated GQ unfolding does not require ATPase and helicase activity, we performed measurements with a mutant BLM construct that cannot hydrolyze ATP, BLM^K695M^. This construct is the core BLM with a lysine to methionine substitution at amino acid 695, and is similar to BLM^K695A^ and BLM^K695T^ mutants studied earlier (54,62). The absence of helicase activity of BLM^K695M^ was confirmed by the absence of DNA unwinding activity with the forked DNA construct (see Supplementary Figure S7A for a schematic of the construct) at saturating BLM^K695M^ (500 nM) and ATP concentration (1 mM). Despite the lack of helicase activity, BLM^K695M^ showed significant GQ unfolding activity in the absence of ATP (Supplementary Figures S9 and S10). These measurements confirm that BLM does not require ATP hydrolysis to unfold GQ.

### BLM-mediated GQ unfolding is most efficient in ATPγS state and least efficient in ADP state

Figure 2 shows data from smFRET studies on pd-hGQ12T in which the BLM concentration is kept at 1 μM while ATPγS (Figure 2A–D) or ADP (Figure 2E–H) is titrated. In Figure 2A–D, it is clear that increasing the ATPγS concentration increases the low FRET population, suggesting that BLM-mediated GQ unfolding is more efficient in the ATPγS state compared to the nt-free state. Figure 2D shows results of subtraction analysis in which the zero ATPγS, nt-free and 1 μM BLM state (Figure 1D) is used as the reference state. The Langmuir binding isotherm fit in Figure 2D results in *K*_eq_ = 4.2 ± 1.1 μM (for ATPγS) and *α* = 30 ± 4%. The relatively low *K*_eq_ obtained suggests that the system reaches saturation at very low ATPγS concentration. The *α* parameter of this fit is a measure of the difference in the efficiency of BLM-mediated GQ unfolding in the ATPγS state relative to the nt-free state (Figure 1D). In this case, 30% more unfolding is observed in the presence of ATPγS relative to its absence at 1 μM BLM. These results are also consistent with the data presented in Figure 1. The unfolded population for 1 μM BLM in the nt-free state is 40 ± 6% (Figure 1E), while the corresponding value for 1 μM BLM in ATPγS state is 47 ± 3% (Figure 1I). Taking the mean values in these measurements, i.e. 40% and 47%, would result in an 18% increase in the ATPγS state with respect to the nt-free state. Similar measurements were performed with another non-hydrolysable ATP analog, AMP-PNP. In agreement with the ATPγS results, BLM-mediated GQ unfolding was more efficient in the AMP-PNP state compared to the nt-free state. An analysis similar to that shown in Figure 2D resulted in *α* = 18 ± 1% and *K*_eq_ = 2.1 ± 0.3 μM for AMP-PNP titration in 1 μM BLM (Supplementary Figure S11).

Figure 2E–H show an interesting trend in BLM-mediated GQ unfolding as ADP is titrated in the presence of 1 μM BLM. Increasing ADP concentration increases the folded GQ conformation as opposed to the unfolded population, suggesting that BLM is less efficient in unfolding GQ in the ADP state compared to the nt-free state. In order to quantify this, the increase in the folded population is obtained from a subtraction analysis using the nt-free state as the reference, e.g. zero ADP and 1 μM BLM concentration (Figure 1D). A Langmuir isotherm fit to the data results in *K*_eq_ = 1.2 ± 0.1 μM (for ADP) and *α* = 14 ± 1%. The low *K*_eq_ suggests that BLM-mediated GQ unfolding is very sensitive to ADP concentration and even a low micromolar concentration of ADP is adequate to reduce the unfolding activity by as much as 14%. Langmuir binding isotherm fit constants for Figure 2 are listed in Table 2. These results are also consistent with those obtained in Figure 1 within the uncertainties of the measurements. The data in Figure 1M show 30 ± 3% BLM-mediated GQ unfolding at 1 μM BLM in 1 mM ADP compared to 40 ± 7% at 1 μM BLM in nt-free state (Figure 1E). Comparing the mean values in these measurements, 30% and 40%, would result in a 25% reduction in BLM-mediated GQ unfolding in ADP state with respect to the nt-free state. The smFRET histograms and the subtraction analysis for ATPγS and ADP concentrations not shown in Figure 2, are shown in Supplementary Figures S12 and S13.

The BLM footprint on ssDNA has been reported as either 7 (63) or 14 nt (64). To ensure that our results obtained with the GQ substrate containing a 12 nt overhang are not compromised by an inefficient binding of BLM to this overhang, we measured BLM-mediated GQ unfolding in different nucleotide states on pd-hGQ15T at saturating BLM concentrations. pd-hGQ15T is a GQ construct with 15-nt long 3′ overhang which is formed by annealing hGQ15T and the RNA-Stem given in Table 1. These measurements yielded consistent results with those on pd-hGQ12T and showed that BLM-mediated GQ unfolding is most efficient in the ATPγS state, followed by the nt-free state and then the ADP state (Supplementary Figure S14).

### BLM-GQ interactions with ATP

**Figure 3.**
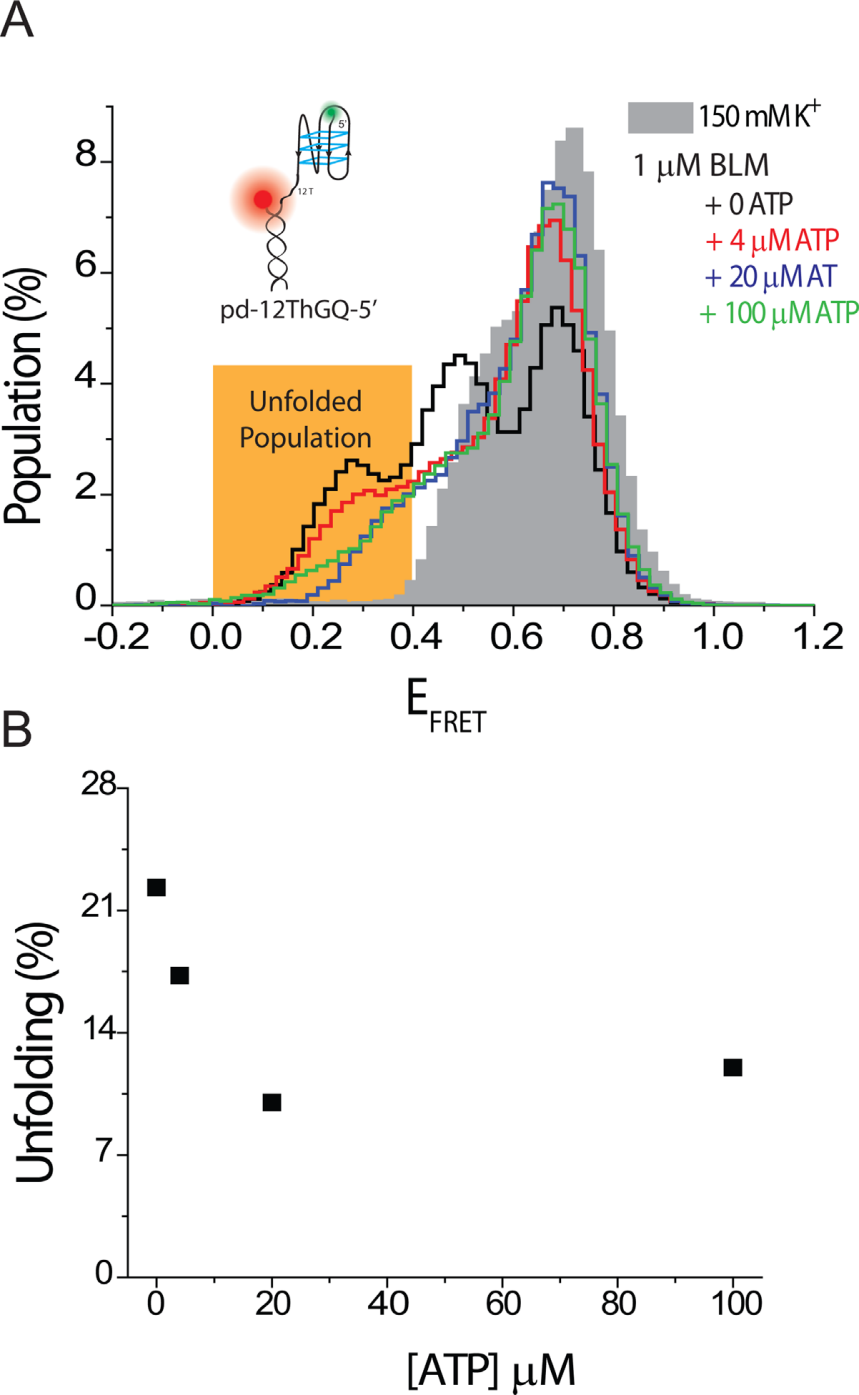
smFRET measurements on pd-12ThGQ-5′ construct at 150 mM K^+^ and different ATP concentrations. The BLM concentration is maintained at 1 μM during ATP titration. (A) The gray shaded histogram shows the folded state, while the histogram with black, red, green and blue outlines represent 0, 4, 20 and 100 μM ATP, respectively. The unfolded GQ population is represented by E_FRET_ ≤ 0.40, and is highlighted by the orange rectangle. The peak at E_FRET_ ≈ 0.50 in black histogram represents the BLM-bound folded GQ state. The inset shows a schematic of the DNA construct used for these measurements. (B) The total unfolded population in these steady-state histograms, as obtained by integrating the area at E_FRET_ ≤ 0.40, decreases as ATP concentration is increased. This decrease is attributed to more frequent dissociation of BLM from the DNA substrate.

We also measured BLM-mediated GQ unfolding in the presence of ATP with pd-hGQ12T. The measurements were performed at 150 mM K^+^ and pH 7.5. However, we observed that BLM not only unfolds the GQ but also unwinds the RNA-DNA heteroduplex stem at significant levels at saturating BLM and ATP concentrations (Supplementary Figure S9B). To avoid complications that would arise due to duplex unwinding, we repeated these measurements with a DNA construct in which the polarity of DNA was reversed, e.g. 5′ is the free end, and the overhang that enables BLM binding is placed between the duplex and GQ (Figure 3 and Table 1, pd-12ThGQ-5′ construct). With this construct, BLM translocates away from the duplex, therefore, unwinding of the duplex is avoided. These measurements clearly showed that BLM unfolds intramolecular GQ in an ATP-dependent manner, however, an interesting trend is observed in the steady-state histograms (Figure 3). In this figure, the unfolded GQ population is represented with the peak at E_FRET_ ≈ 0.25, while the peak at E_FRET_ ≈ 0.50 represents the BLM-bound folded GQ state. With increasing ATP concentration at 1 μM BLM, the unfolded GQ population systematically decreases, with maximum unfolding taking place in the absence of ATP, e.g. nucleotide-free state. This trend is similar to that observed for ADP with pd-hGQ12T, which suggests that more frequent dissociation of BLM from the DNA enables refolding of the GQ and therefore a decrease in the unfolded GQ population. However, the kinetics of BLM-GQ interactions is not captured in these steady-state histograms. It is possible that GQ repetitively unfolds and refolds at a rate that increases with ATP concentration, as recently reported for Pif1 helicase (65). The steady-state histograms do not detect these types of underlying dynamics which are crucial for attaining an accurate picture of BLM-GQ interactions in the presence of ATP. A comprehensive study of these dynamics is beyond the scope and focus of this manuscript.

### BLM-mediated GQ unfolding correlates with stability of BLM binding to the ssDNA overhang

**Figure 4.**
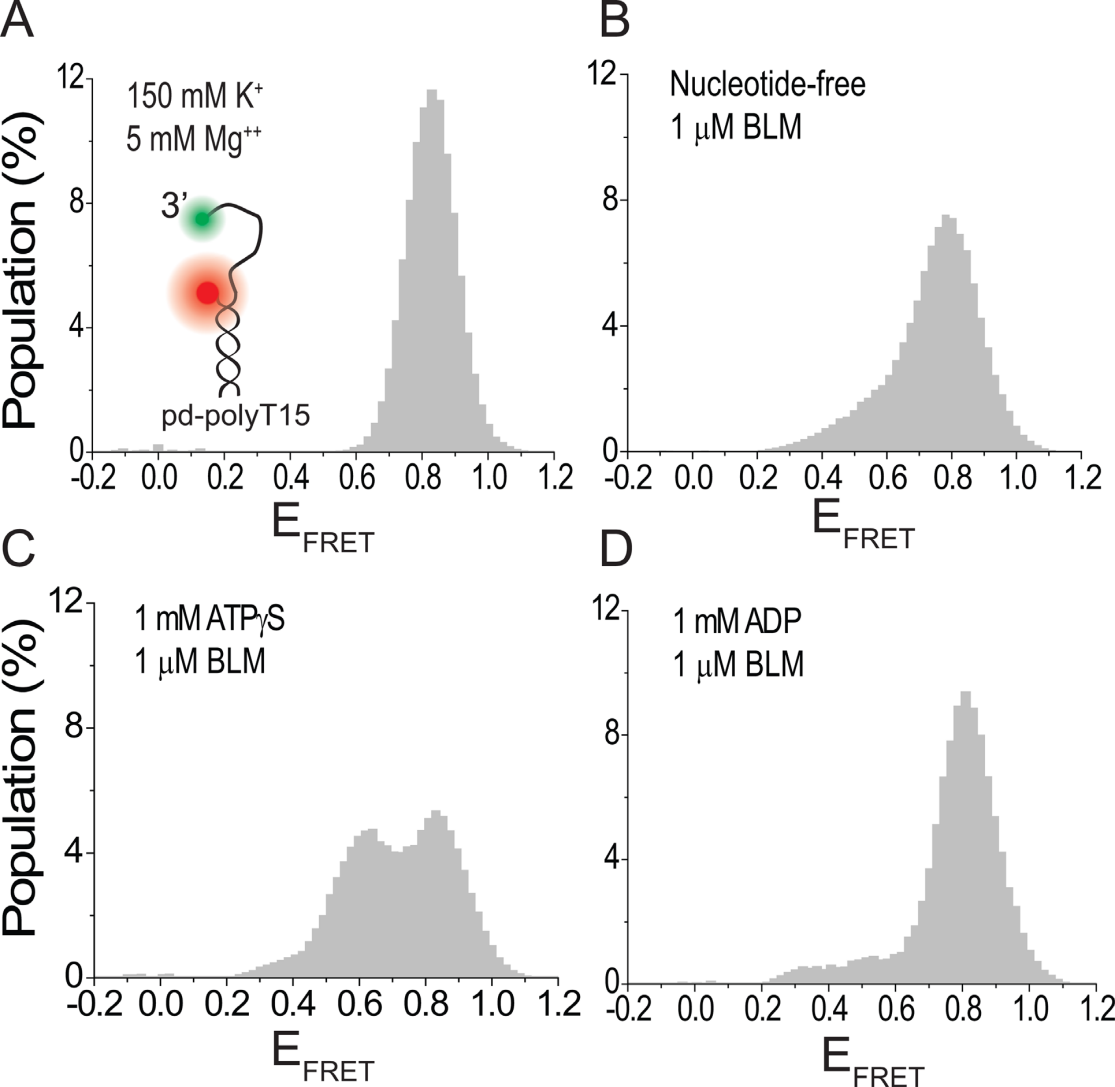
smFRET measurements on pd-polyT15 (pdDNA with 15 thymine long 3′ overhang) at 150 mM K^+^. BLM binding to ssDNA representing the overhang in the GQ substrate depends on the nucleotide state. (A) pd-polyT15 is in coiled conformation with E_FRET_ ≈ 0.85 before BLM is added. (B)–(D) BLM binding to the overhang in different nucleotide states at 1 μM BLM. (B) Nt-free state. (C) 1 mM ATPγS. (D) 1 mM ADP. Binding is most efficient in the ATPγS state, followed by the nt-free state and then the ADP state.

The data presented thus far show that BLM-mediated GQ unfolding is most efficient in the ATPγS state, followed by the nt-free and ADP states. Earlier bulk measurements have shown that the stability of BLM binding to ssDNA in different nucleotide states follows a similar order (63). This correlation suggests that BLM-mediated GQ unfolding strongly depends on the stability of BLM binding to the overhang ssDNA in the vicinity of GQ. In order to study binding of BLM to an overhang ssDNA in the absence of GQ, we performed smFRET measurements on pd-polyT15 which is formed by hybridizing polyT15 and the RNA-Stem (Table 1). pd-polyT15 has a 15-nt long flanking 3′-ssDNA which is same in length and sequence to the overhang of pd-hGQ15T. A scheme of the construct is given in the inset of Figure 4A. BLM binding to pd-polyT15 results in a lower E_FRET_ compared to the coiled state of DNA, and enables us to quantify BLM binding activity. For these measurements, all experimental conditions were kept identical to those used for the GQ constructs (150 mM K^+^, pH 7.5). Figure 4A shows the smFRET histogram for the coiled state which shows a single peak at E_FRET_ ≈ 0.85. Figure 4B–D show smFRET histograms when 1 μM BLM is added to the chamber in nt-free, 1 mM ATPγS and 1 mM ADP states, respectively. The new peak at E_FRET_ ≈ 0.60 represents the DNA population that is bound by BLM. A subtraction analysis performed on these data with the coiled state in Figure 4A taken as the reference shows that 21%, 36% and 12% of DNA molecules are bound by BLM in the nt-free, ATPγS and ADP states, respectively. Thus, the efficiency of BLM binding to ssDNA in different nucleotide states and the efficiency of BLM-mediated GQ unfolding in these nucleotide states show the same trend. A quantitative comparison of BLM binding to pd-polyT15 and BLM unfolding of pd-hGQ15T in different nucleotide states shows significant variation: 21%, 36% and 12% for binding in nt-free, ATPγS and ADP states, respectively, for pd-polyT15, compared to 48%, 57% and 22% unfolding in the respective nucleotide-state for the GQ construct (Supplementary Figure S14). This difference suggests that once pd-hGQ15T unfolds, the unfolded conformation is maintained for extended time periods before it can refold.

### BLM-mediated GQ unfolding increases with the overhang length

**Figure 5.**
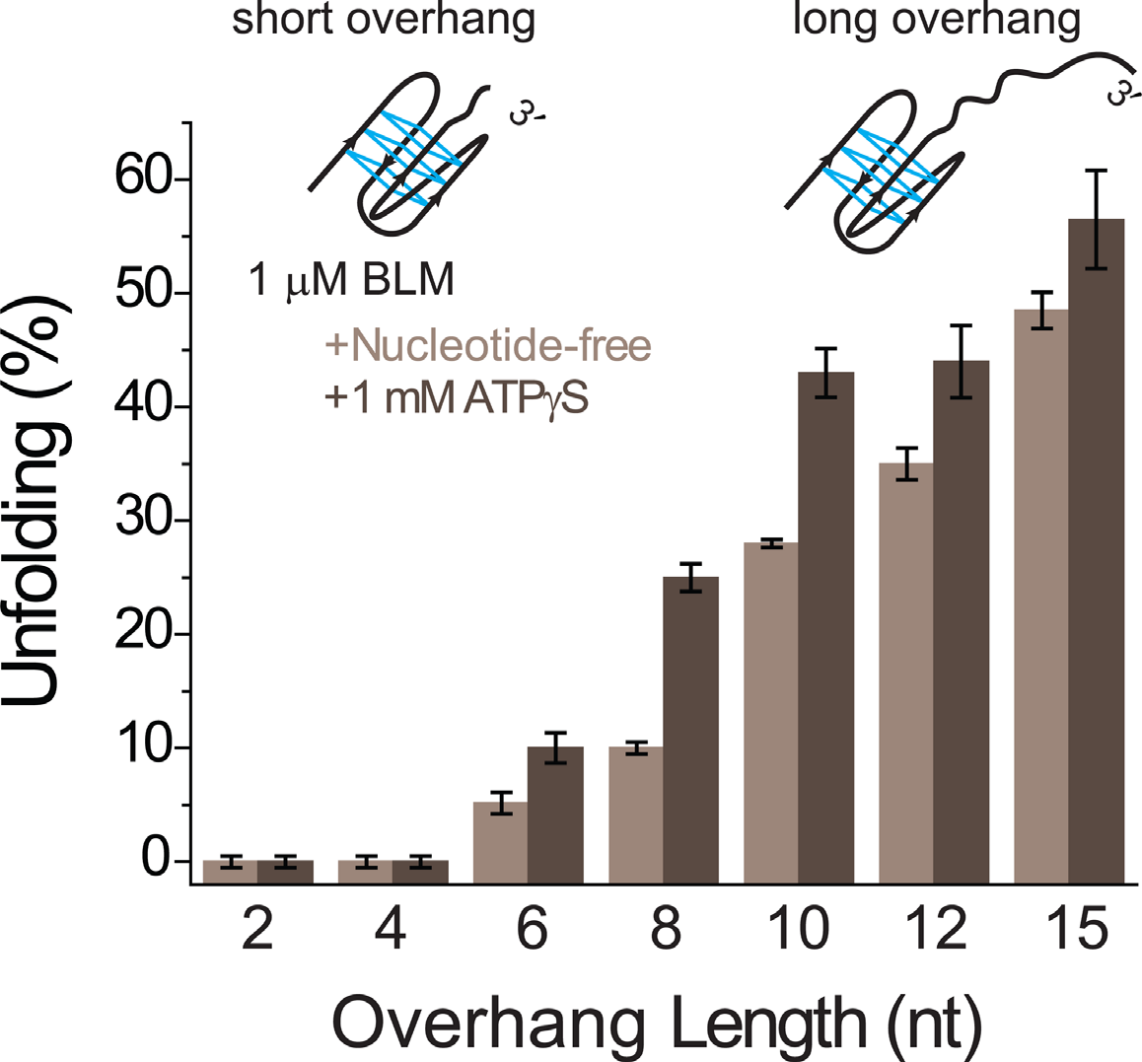
Bar diagrams illustrating the overhang length dependence of BLM-mediated GQ unfolding in the nt-free (light gray) and 1 mM ATPγS states (dark gray). All measurements were carried out at 150 mM K^+^, and 1 μM BLM concentration. Overhangs of 2, 4, 6, 8, 10, 12 and 15 nt were studied. The unfolded populations were determined by subtracting the folded state at 150 mM K^+^ for each construct from the respective nucleotide state with 1 μM BLM concentration. The error bars were obtained based on the variation in BLM-mediated GQ unfolding activity for different data sets.

In order to systematically study the influence of the ssDNA overhang in GQ substrates on BLM-mediated GQ unfolding, we performed smFRET studies in which the overhang length was systematically varied. Increasing the overhang length is expected to result in higher BLM-binding stability and in turn, higher BLM-mediated GQ unfolding. Figure 5 shows results for 2, 4, 6, 8, 10, 12 and 15 nt long overhangs in nt-free and 1 mM ATPγS states. pd-hGQxT with *x* = 2, 6, 8, 10, 12 or 15 and pd-hGQ4nt DNA constructs were used for these studies (see Table 1 for sequences). The BLM concentration was kept at 1 μM and the ion concentration was 150 mM K^+^ for all experiments. The bars shown in Figure 5 were obtained from subtraction analysis in which the folded state, in the absence of BLM or ATPγS, was taken as the reference. We observed a systematic increase in the unfolding activity as the overhang length increased between 6 and 15 nt. We did not observe any unfolding for 2 and 4 nt long overhangs, suggesting that a minimum overhang length of 5–6 nt is required for BLM-mediated GQ unfolding. This result is similar to that obtained for intermolecular GQs which required a 4 nt overhang for BLM-mediated GQ unfolding (52). For the constructs that showed BLM-mediated GQ unfolding, the ATPγS state consistently showed 10–60% higher activity compared to the nt-free state.

### BLM-mediated GQ unfolding depends on the stability of GQ

All our work on BLM-GQ interactions reported so far was performed at 150 mM K^+^ in order to mimic the physiological conditions. The K^+^ concentration is an important determinant of the GQ stability and folding conformation (15), both of which could significantly influence the interactions between BLM and GQ. In order to test whether BLM-mediated GQ unfolding depends on the stability of GQ, we performed smFRET measurements at 50 mM K^+^, which would reduce GQ stability (66) and increase BLM binding stability to the overhang. BLM was titrated in the nt-free state and ATPγS was titrated in the presence of 300 nM BLM at 50 mM K^+^ (see Supplementary Figures S15 and S16). BLM titration in the nt-free state resulted in *α* = 55 ± 4% and *K*_eq_ = 28 ± 9 nM. Note that the *K*_eq_ at 50 mM K^+^ is 10.8-fold lower than that at 150 mM K^+^ (Figure 1E and Table 2). In order to quantify the increase in BLM binding affinity to ssDNA due to reduced ion concentration, we measured the equilibrium constant in 50 and 150 mM Na^+^ in the nt-free state (Supplementary Figure S17). The protein stock used for these measurements was in Na^+^, therefore, the measurements were performed in Na^+^ rather than K^+^. As BLM binding to ssDNA should not depend on the type of monovalent ion, this change is not expected to influence the results. We used a pdDNA with a 15-nt long 3′ ssDNA tail (pd-polyT15). These measurements showed ∼4.7× increase in *K*_eq_ in 150 mM Na^+^ compared to 50 mM Na^+^. As we observed a 10.8× decrease in *K*_eq_ in 50 mM K^+^, we conclude that the decrease in GQ stability contributes ∼2.3-fold to the increase in the *K*_eq_ in 150 mM K^+^. These results demonstrate that both BLM binding affinity and GQ stability are significant contributors to the change in the equilibrium constant for BLM-mediated GQ unfolding. Finally, we measured BLM-mediated GQ unfolding as a function of ATPγS concentration in 300 nM BLM at 50 mM K^+^. These measurements resulted in *α* = 23% and *K*_eq_ = 0.7 ± 0.1 μM, which is 6-fold lower compared to *K*_eq_ at 150 mM K^+^ (Figure 2D).

**Figure 6.**
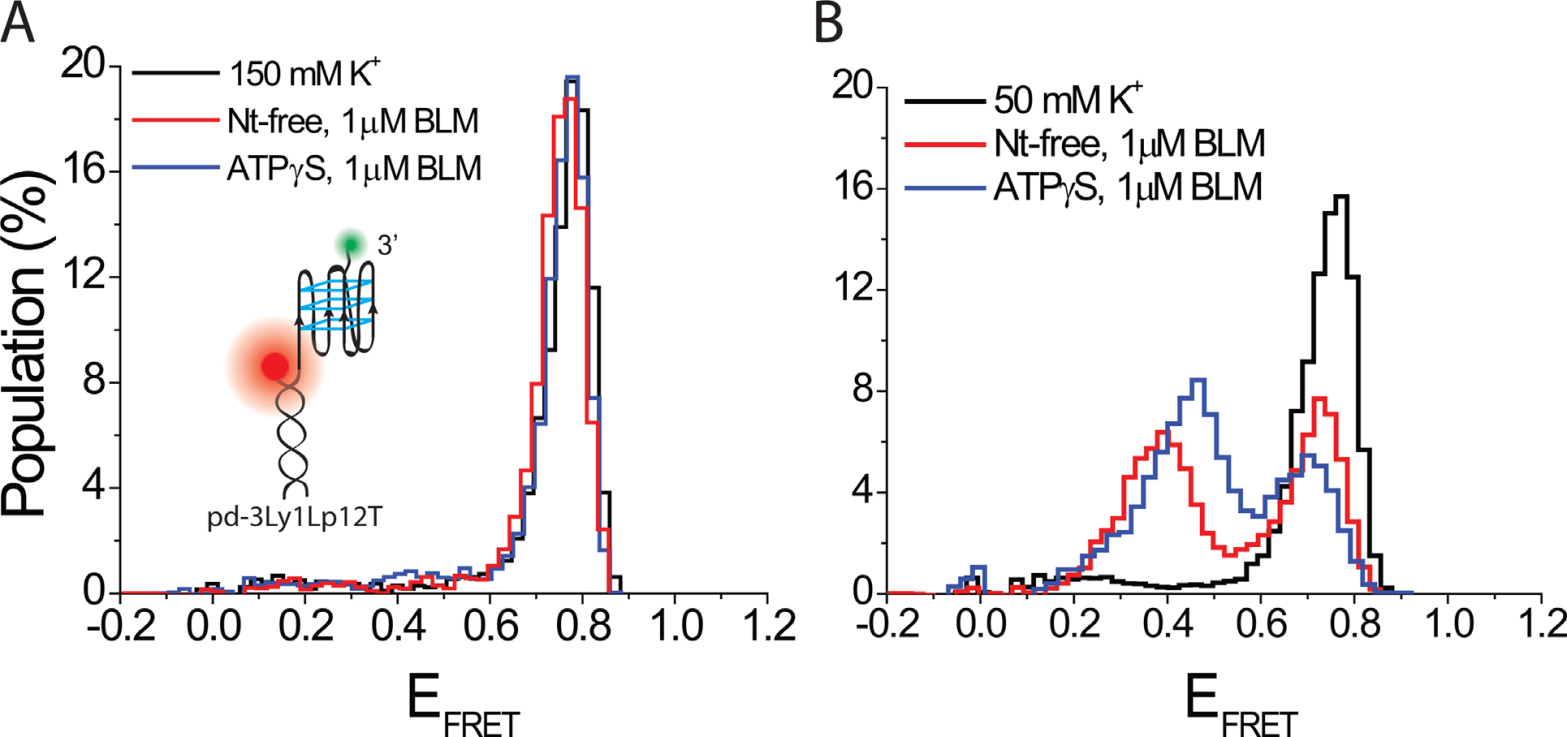
smFRET histograms showing BLM-mediated unfolding for pd-3Ly1Lp12T, three layer one loop GQ construct with 12 nt long 3′ overhang. (A) BLM cannot unfold this GQ at 150 mM K^+^ in either nt-free or 1 mM ATPγS states. These results show that the ability of BLM to unfold intramolecular GQ in the absence of ATP under physiological ionic conditions is substrate specific. (B) Significant GQ unfolding is observed in both nt-free and ATPγS states when ion concentration is reduced to 50 mM K^+^, in which GQ is less stable and BLM binding to the overhang is more efficient.

Another method to modulate GQ stability is to change the length of the loops in the GQ structure. Shortening the loops is known to increase thermal stability of GQ (67) as well as increasing the stability of GQ against RPA-mediated unfolding (43). RPA is a ssDNA binding protein and does not require ATP for unfolding GQ, making it a similar system to BLM studies in the absence of ATP. In order to test BLM-mediated GQ unfolding on a GQ construct significantly more stable than the telomeric GQ structures used in this study, a three layer GQ with single nucleotide loops and an overhang of 12 thymines was used (will be referred to as pd-3Ly1Lp12T). To illustrate, this construct was shown to have two orders of magnitude higher stability against RPA-mediated unfolding compared to the human telomeric GQ with the same overhang (43). Our measurements with pd-3Ly1Lp12T at 150 mM K^+^ did not show any unfolding at 1 μM BLM in either nt-free or 1 mM ATPγS states (Figure 6A), in agreement with the increased stability of this construct. However, our measurements at 50 mM K^+^ showed BLM-mediated unfolding of pd-3Ly1Lp12T in both nt-free and ATPγS states. Figure 6B shows folding of pd-3Ly1Lp12T, and its BLM-mediated unfolding in the nt-free and ATPγS states at 50 mM K^+^. At 1 μM BLM, we observed 46% unfolding in nt-free state, and 58% unfolding at 1 mM ATPγS, showing a similar ATPγS enhancement of BLM-mediated GQ unfolding as with the other constructs. This experiment shows that the nucleotide-independent BLM-mediated unfolding of the pd-hGQ12T intramolecular GQ at 150 mM K^+^ is substrate specific. Such an activity cannot be achieved with more stable GQ, such as pd-3Ly1Lp12T, unless a significant reduction is made in the monovalent ionic strength.

### Directionality of the overhang impacts BLM binding and its GQ unfolding activity

**Figure 7.**
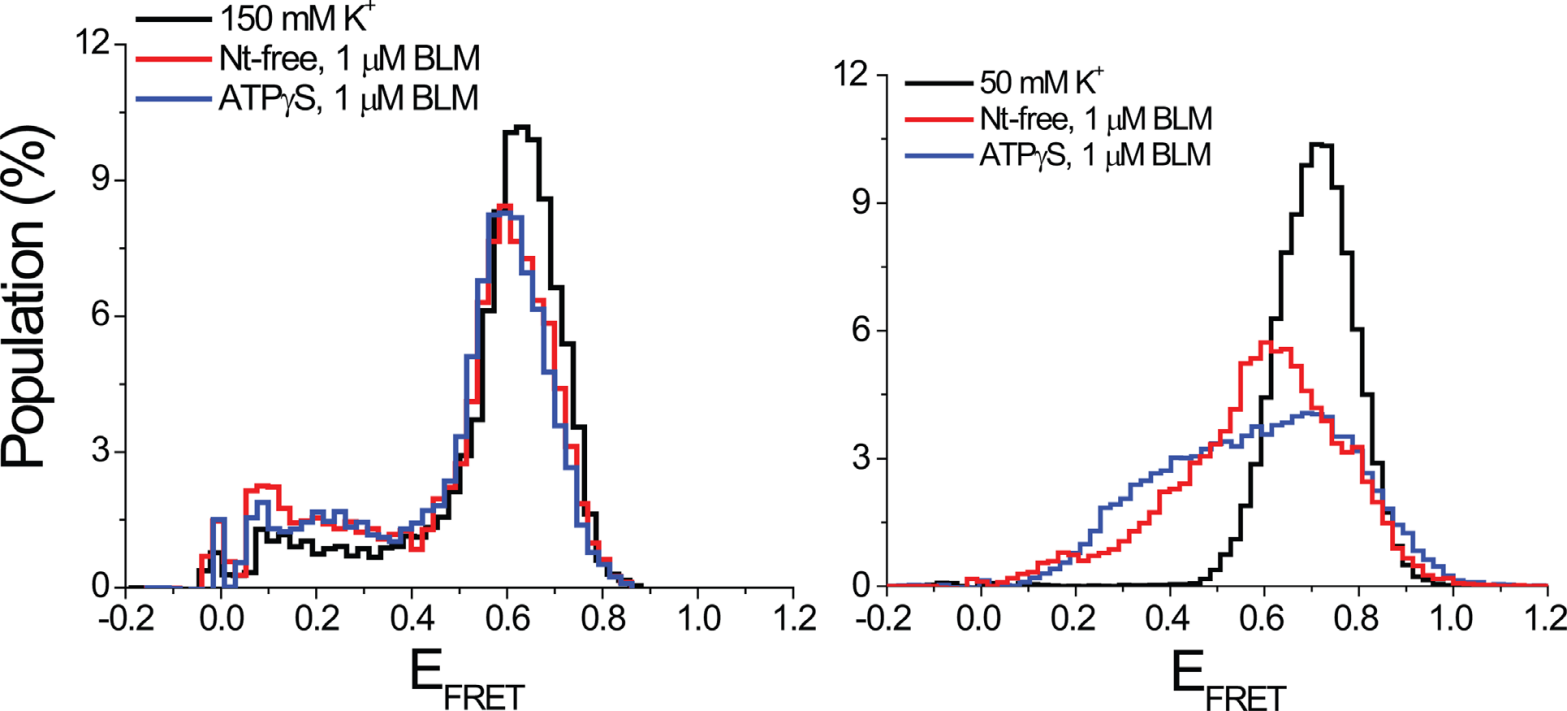
smFRET histograms showing BLM-mediated GQ unfolding of pd-hGQ12T-5′. This construct has a 12 thymine long 5′ overhang. (A) BLM cannot unfold this construct at 150 mM K^+^ in either nt-free or ATPγS states, indicating orientation of binding on the overhang is important. (B) However, if the GQ stability is lowered at 50 mM K^+^, significant BLM-mediated GQ unfolding occurs in both nt-free and ATPγS states.

BLM translocates in the 3′ to 5′ direction and, therefore, binds to the overhang ssDNA in an orientation that accommodates this directionality. As BLM is able to unfold GQs in the absence of ATP, presumably without translocating on DNA, we sought to determine whether the polarity of the overhang is still significant for BLM-mediated GQ unfolding. For these studies, we used a construct identical to pd-hGQ12T with the exception of the overhang having a 5′-end rather than a 3′-end (will be referred to as pd-hGQ12T-5′). BLM-mediated GQ unfolding was not observed in this construct at 150 mM K^+^ in either nt-free or ATPγS states even at 1 μM BLM concentration (Figure 7A). The low FRET population observed upon adding BLM to the chamber is not significantly different from the folded state at 150 mM K^+^, hence, we do not consider this as BLM-mediated GQ unfolding. However, at 50 mM K^+^, 21% and 30% unfolding were observed in nt-free and ATPγS states, respectively (Figure 7B), demonstrating that the ionic strength mediated effects on GQ stability and BLM binding to the 5′ overhang likely influence this outcome. To further explore these points, we studied BLM binding to a pdDNA with a 15-thymine ssDNA overhang that has a 5′ end (will be referred to as pd-polyT15–5′). pd-polyT15–5′ is formed by hybridizing polyT15–5′ and DNA-Stem strands given in Table 1. smFRET studies on pd-polyT15–5′ at 150 mM K^+^ did not show any BLM binding in the nt-free state and showed very little binding in the ATPγS state (Supplementary Figure S18A) in contrast to the significant binding (36%) observed for the corresponding 3′ construct (pd-polyT15 construct in Figure 4) in the ATPγS state (Supplementary Figure S18A). Reducing the K^+^ concentration to 50 mM resulted in significantly higher BLM binding to pd-polyT15–5′ in the ATPγS state (53%) even though the nt-free state still did not show significant BLM binding. Interestingly, BLM-mediated unfolding of pd-hGQ12T-5′ was observed in the nt-free state at 50 mM K^+^ in the presence of 1 μM BLM (Figure 7B), even though significant BLM binding to pd-polyT15–5′ was not observed under these conditions (Supplementary Figure S18B). These two observations may be reconciled by more efficient BLM binding to the 35-nt long ssDNA that becomes available upon transient melting of the pd-hGQ12T-5′ GQ compared to the 15 nt in pd-polyT15–5′. Such transient melting events could either be induced by BLM or by thermal fluctuations. These observations are consistent with the lower GQ unfolding activity observed for 5′-end construct being influenced by the lower binding ability of BLM to this overhang.

### GQ conformation dependence of BLM-mediated GQ unfolding

**Figure 8.**
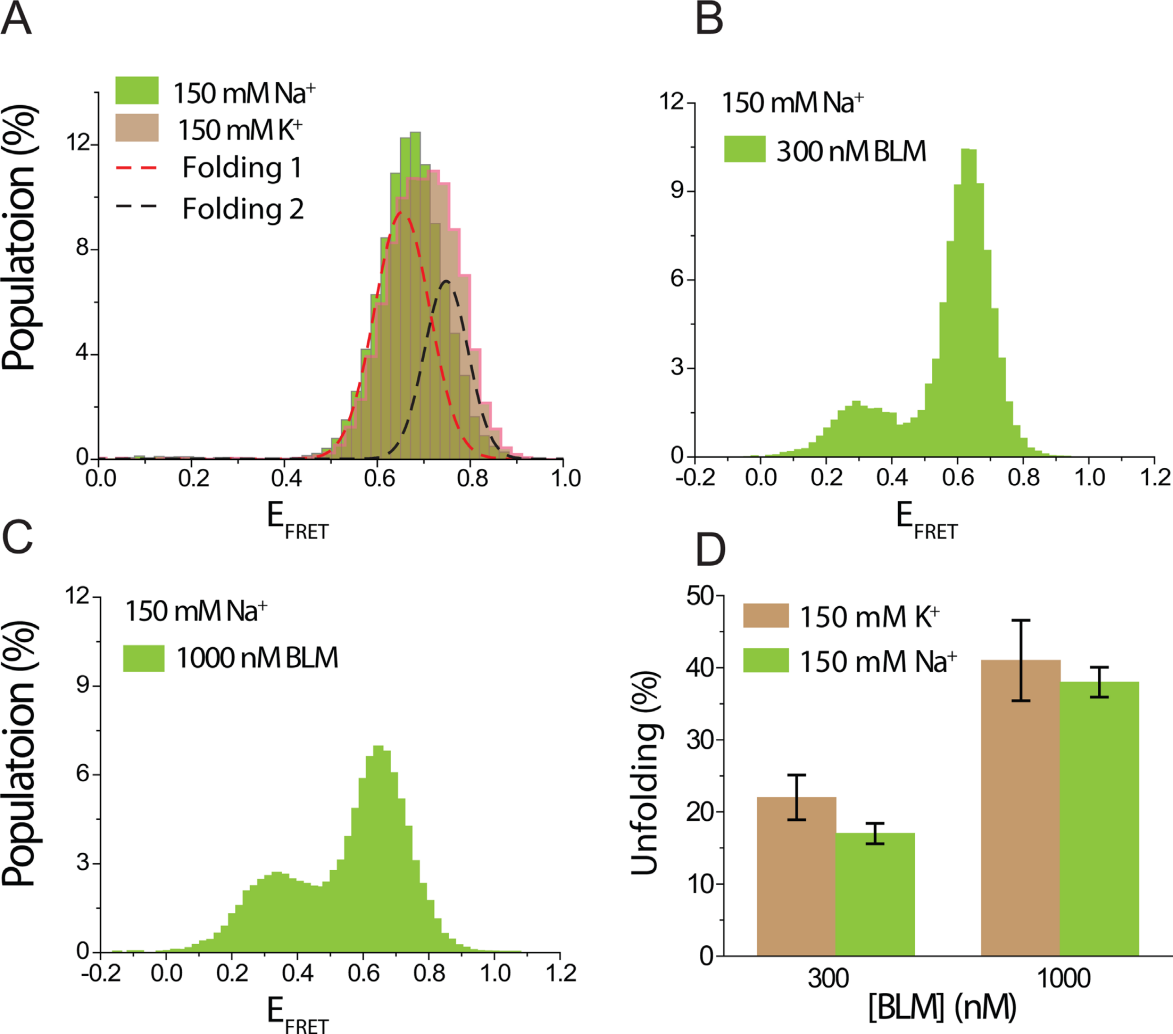
A comparison of BLM-mediated GQ unfolding in 150 mM K^+^ and 150 mM Na^+^, in which pd-hGQ12T folds into different conformations. (A) The green shaded histogram represents the folded GQ state in 150 mM Na^+^, and the brown histogram represents the folded state in 150 mM K^+^. The histogram in Na^+^ is narrower and can be fit by a single Gaussian peak, indicative of a single conformation, while that for K^+^ is broader and requires two Gaussian peaks for fitting (shown by black and red dashed curves), indicative of multiple folding conformations. (B) and (C) BLM-mediated GQ unfolding in 150 mM Na^+^ in 300 and 1000 nM BLM, respectively. (D) A comparison of BLM-mediated GQ unfolding in 150 mM Na^+^ and 150 mM K^+^ for 300 and 1000 nM BLM. These data show that unfolded GQ population is at similar levels in both ionic conditions, suggesting that BLM unfolds different conformations at similar levels.

GQs often fold into multiple conformations in K^+^. Changing the loop lengths, number of layers or the overhang sequences could result in changes in conformations (68) in addition to variations in the stability of the GQ. In particular, human telomeric sequence has been shown to fold into parallel, anti-parallel and various hybrid conformations in 150 mM K^+^, while it folds into a single anti-parallel conformation in 150 mM Na^+^ (61). In order to study a possible conformation dependence of BLM-mediated GQ unfolding while maintaining similar DNA binding affinities, we compared BLM-mediated GQ unfolding in 150 mM K^+^ with that in 150 mM Na^+^ for pd-hGQ12T. As shown in Figure 8A, pd-hGQ12T shows a single narrow smFRET peak in Na^+^, which is indicative of a single conformation, unlike the broader peak in K^+^, which is indicative of multiple conformations (shown with black and red dashed curves). Figure 8B and C show BLM-mediated unfolding at 300 and 1000 nM BLM, respectively, in 150 mM Na^+^. Figure 8D shows a comparison of the unfolded GQ populations for 300 and 1000 nM BLM in 150 mM Na^+^ and 150 mM K^+^. Note that 22 ± 3% and 17 ± 2% GQ were unfolded by 300 nM BLM in 150 mM K^+^ and 150 mM Na^+^, respectively. Similarly, 41 ± 6% and 38 ± 2% GQ were unfolded by 1000 nM BLM in 150 mM K^+^ and 150 mM Na^+^, respectively. Therefore, BLM unfolds GQ at similar levels, within the uncertainties of the measurements, in Na^+^ and K^+^. These results suggest that BLM unfolds different conformations of GQ with similar efficiency.

### Protein-mediated GQ unfolding for other RecQ family helicases

**Figure 9.**
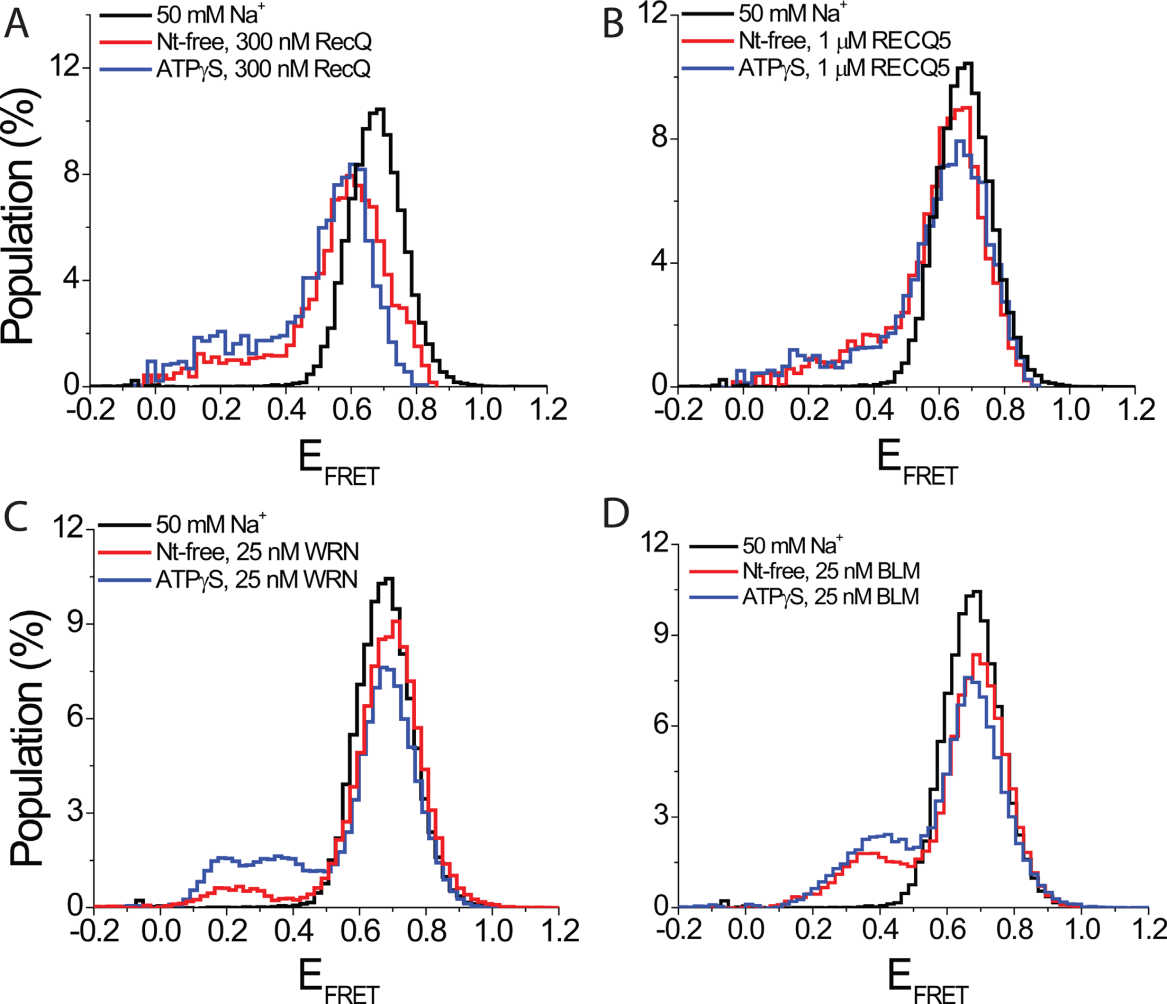
smFRET histograms showing interactions of *E. coli* RecQ, human RECQ5, human WRN and BLM with pd-hGQ12T at 50 mM Na^+^. Note the cation was changed to Na^+^ as GQ unfolding was not observed in K^+^, which results in a more stable GQ, for RecQ and RECQ5. (A) Signs of GQ destabilization are observed in both the nt-free and ATPγS states in 300 nM RecQ. (B) 1 μM RECQ5 also shows weak GQ unfolding activity in both nt-free and ATPγS states. (C) WRN unfolds GQ even at 25 nM concentration in both nt-free and ATPγS states. (D) As a reference to compare with WRN-mediated GQ unfolding, BLM-mediated GQ unfolding is also measured in 25 nM BLM at 50 mM Na^+^. The observed GQ unfolding activities of BLM and WRN are similar under these conditions.

In order to determine whether BLM is an exceptional case in terms of a helicase destabilizing GQ in the absence of ATP, we sought to test whether such an activity is observed for closely related proteins from the RecQ family. *E. coli* RecQ, human RECQ5 and human WRN, all full-length constructs, were used for these studies. WRN has been shown to unfold GQ in the presence of ATP (53). We are not aware of any studies on RecQ and RECQ5 in terms of their GQ unfolding activity. pd-hGQ12T construct was used for these studies. RecQ and WRN studies were performed at 300 and 25 nM concentrations, respectively, due to limitations in the protein stock concentrations. The RECQ5 measurements were performed at 1 μM concentration, similar to BLM. Due to these lower protein concentrations, we performed measurements under different ionic conditions that are more likely to demonstrate protein-mediated GQ unfolding. We also performed these measurements in nt-free and ATPγS states in order to check the consistency of BLM results, i.e. higher GQ unfolding in ATPγS state, with these proteins. In the case of RecQ, we performed measurements in 50 mM K^+^ or 50 mM Na^+^. RecQ did not show any GQ unfolding activity in 50 mM K^+^ (see Supplementary Figure S19A) while signs of GQ destabilization were observed at 50 mM Na^+^ in both nt-free and ATPγS states (Figure 7A). As BLM binding to ssDNA is expected to be similar for 50 mM K^+^ and 50 mM Na^+^, the observed difference in BLM-mediated GQ unfolding is considered to be due to the weaker GQ stability in Na^+^ (69). In both 50 mM K^+^ and 50 mM Na^+^, the folded FRET peak shifts from E_FRET_ ≈ 0.68 to E_FRET_ ≈ 0.52 upon introduction of RecQ. This lower peak has very small population at E_FRET_ < 0.40, which is the expected range for the unfolded DNA of this length (Supplementary Figure S1). Therefore, the shift of the FRET peak position upon adding RecQ is most likely due to binding of one or more RecQ proteins to the 12-nt long overhang, rather than unfolding of the GQ. Similarly, RECQ5 did not unfold GQ at 150 mM K^+^ (see Supplementary Figure S19B), but showed GQ unfolding activity at 50 mM Na^+^ in both nt-free and ATPγS states (Figure 9B). Due to the very low stock concentration, measurements with WRN were performed only at 50 mM Na^+^. Nevertheless, significant GQ unfolding was observed even at 25 nM WRN (Figure 9C). In order to perform a quantitative comparison with BLM, similar measurements were performed with 25 nM BLM at 50 mM Na^+^ (Figure 9D). At 50 mM Na^+^, 25 nM WRN resulted in 8% and 25% unfolding of pd-hGQ12T in nt-free and ATPγS states, respectively. Similarly, 25 nM BLM resulted in 18% and 25% unfolding of pd-hGQ12T in nt-free and ATPγS states, respectively. Combined, our measurements on RecQ, RECQ5 and WRN show a broad range of efficiencies in GQ unfolding activity in the absence of ATP, which have implications for the role of DNA binding activity versus translocase activity of helicases in GQ destabilization.

## DISCUSSION

Based on the presented data, we propose that BLM-mediated GQ unfolding strongly depends on the stability of BLM binding in the vicinity of GQ and does not require any nucleotide cofactor. The binding stability is primarily affected by the length of the 3′ overhang ssDNA and the nucleotide state. Prior bulk biochemical studies have shown that BLM binding to ssDNA, of similar length to the overhangs used in our study, is significantly more stable in the nt-free and AMP-PNP states compared to the ADP state and that BLM dissociates from ssDNA in the ADP state (63,64). These observations are also complemented by our smFRET measurements which show that binding of BLM to pd-polyT15, a pdDNA with a 15-nt long ssDNA overhang, is most stable in the ATPγS state, followed by the nt-free state and then the ADP state. The enhanced GQ unfolding by BLM in the presence of non-hydrolysable ATP analogs ATPγS and AMP-PNP demonstrate the role of nucleotide binding in BLM-mediated GQ unfolding. This enhanced activity suggests that translocation of the helicase is not required and is certainly not the only activity that destabilizes the GQ.

Comparison of the *α* parameter of Langmuir binding analysis indicates that under saturating BLM and nucleotide concentrations, BLM-mediated GQ unfolding is 10–60% more efficient in the ATPγS state and 15–30% less efficient in the ADP state compared to the nt-free state (Table 2). The maximum GQ unfolding does not rise above 55% in any nucleotide state suggesting a dynamic equilibrium between BLM-mediated GQ unfolding and GQ refolding. The equilibrium constants describing BLM-mediated GQ unfolding for different nucleotide states show large variations. BLM titration in the nt-free state results in *K*_eq_ = 305 ± 16 nM, while *K*_eq_ = 108 ± 24 nM for the ATPγS state, and *K*_eq_ = 54 ± 5 nM for ADP state. These concentrations are significantly higher than the dissociation constants reported for BLM (63,64) as they are a measure of not only BLM binding to the overhang ssDNA but also of unfolding of the GQ that follows this binding. On a related note, our method of probing BLM-mediated GQ unfolding has an advantage over the other published method in which GQ is essentially prevented from refolding due to an irreversible transition to a hairpin structure, unless ethylenediaminetetraacetic acid is added to the environment (56). Our data suggest that GQ can refold if not prevented by hairpin formation. This would explain why the maximum unfolding in our assay is less than that of the other study in which about 90% unfolding was observed (56). It might be argued that at high BLM concentration, multiple BLM molecules might bind to ssDNA that becomes available after GQ is unfolded, and in that way essentially create an irreversibly unfolded state. However, smFRET time traces show that such a stable unfolded conformation is not attained even at 1 μM BLM concentration. Supplementary Figure S20 shows that the system remains dynamic with GQ unfolding and refolding multiple times within ∼2 min observation time of our traces.

The correlation between the binding stability of BLM to the overhang and the BLM-mediated GQ unfolding activity highlights the significance of the overhang region in the vicinity of GQ. The function of the overhang region was previously studied in the context of intermolecular GQs and it was shown that BLM unfolds GQ with an overhang as short as 4 nt but cannot unfold GQ that lack an overhang (52). Our results on an intramolecular GQ are similar as we observe unfolding for overhangs of 6 nt or longer. We furthermore demonstrate that the BLM-mediated GQ unfolding efficiency gradually increases as the overhang length is increased. Given the 7 or 14 nt footprint of BLM (63,64), the dramatic increase in BLM-mediated GQ unfolding observed at overhang lengths of 6 and 8 nt could provide supporting evidence for 7 nt footprint. Therefore, localization of BLM to the vicinity of GQ, even if stable binding is not established, as might be the case for 6 nt overhang, enables BLM to interact with the GQ structure and eventually destabilize it. A similar mechanism was suggested for ssDNA binding protein RPA which also unfolds GQs in the absence of ATP (43). These observations are consistent with isothermal differential hybridization (IDH) studies which showed that a protein binding to the vicinity of telomeric GQ dramatically reduces GQ stability as measured by the folding equilibrium constant (*K_F_*) of GQ (70). These IDH studies measured the change *K_F_* via a competition of GQ with Watson–Crick pairing with the complementary C-rich strand. The studies reported an order of magnitude reduction in *K_F_* when a digoxigenin and antidigoxigenin antibody were attached to the vicinity of GQ. To our knowledge, these IDH studies are the first and only work that directly probed the influence of protein binding to the vicinity of GQ and GQ stability. Our studies improve on this important study in several significant ways. In our studies, the dynamic nature of the system is preserved as BLM binding dissociation or GQ folding-unfolding were not trapped in any particular state. In the IDH measurements, the antidigoxigenin antibody is permanently bound to the vicinity of GQ and dsDNA formation is essentially an irreversible step that blocks refolding of the GQ. Furthermore, our studies show that protein-mediated GQ unfolding can be altered by either (i) modulating the binding stability of the protein on the overhang, via changing the nucleotide state or the length of the overhang or (ii) changing the GQ stability, via monovalent ion concentration or changing the loop length in GQ.

Another important issue that should be addressed is whether the truncated BLM construct, RecQ-core of BLM or core-BLM, used in this study is representative of the full-length BLM (wt-BLM) construct that is physiologically relevant. Even though core-BLM has been shown to be a good model system for kinetic studies (12), its DNA binding properties, which are significant for this study, could be different from those of the wt-BLM. There are two main reasons why this study was limited to the core-BLM construct. The first one is a practical reason in that it was not possible to attain high enough wt-BLM concentrations required to observe significant GQ unfolding in the absence of ATP. The lower yield attained due to different requirements of the purification protocol limited protein concentration to ∼50 nM BLM in the sample chamber, which is not high enough to observe wt-BLM-mediated GQ unfolding in our assay conditions which require ≥300 nM core-BLM. The other aspect of this discussion, is the ‘model protein’ aspect of core-BLM. Our study essentially shows that many helicases that bind to the vicinity of GQ have the potential to destabilize these structures in the absence of ATP. In this respect, it is not critical to use the wt-BLM, particularly when core-BLM provides a much broader range of concentrations that enable testing a large array of different cases presented in this work. Even though a detailed kinetic study of BLM-mediated GQ unfolding in ATP and other nucleotide states is missing and would be an excellent complement to this work, the scope of such a study is beyond the current work and the focus is significantly different. Therefore, these topics will be addressed in a future study. To our knowledge, our study is the first to show that BLM-mediated GQ unfolding does not require ATP. However, previous studies were almost exclusively performed on intermolecular GQs. The contrasting results of our study and these earlier works could either arise due to inherent differences between intermolecular and intramolecular GQs or possibly due to better sensitivity of the single-molecule methods we use. In the rare case in which an intramolecular GQ was used, BLM-mediated unfolding of a human telomeric GQ with 10 nt overhang was probed via a bulk fluorescence method in the nt-free state (56). This study did not show the characteristic decrease in the fluorescence signal which was used as a signature for BLM-mediated GQ unfolding. However, we do not think our results necessarily contradict with this study. A novel DNA construct which could transition from a GQ to a hairpin, upon BLM-mediated unfolding of the GQ, was used in that assay (Figure 1A in (56)). The fluorescence intensity of the fluorophore, which is attached to the overhang of GQ, is reduced as a result of hairpin formation, due to its interactions with the complementary strand. A reduction in fluorescence intensity was not observed in the nt-free state even though BLM is shown to bind to the overhang, via polarization measurements. However, in order for a reduction to be observed in fluorescence intensity, BLM needs to unfold the GQ and dissociate from the overhang, which will then allow hairpin formation. If BLM unfolds GQ but does not dissociate from the overhang, the hairpin cannot form and fluorescence intensity would not decrease. Therefore, even if BLM-mediated GQ unfolding takes place, this assay might not be sensitive to it unless BLM dissociates from the overhang. The contrasting results for the intermolecular and intramolecular GQ constructs are significant and might be an indicator of different mechanisms being employed by BLM to interact with these different secondary structures. Therefore, it would be of interest to study whether BLM shows such an unfolding activity in the absence of ATP for other types of DNA secondary structures, such as Holliday junctions or D-loops, using similar single molecule methods. Whether other helicases are also able to unfold intramolecular GQ in the nt-free, ATPγS or ADP states is another important question that is raised by our study. Our measurements on RecQ, RECQ5 and WRN show a broad range of efficiencies in GQ unfolding activity in the absence of ATP. However, recent studies on helicases FANCJ and XPD showed that these helicases can unfold intramolecular and intermolecular GQ, respectively, in the ATP state, but not in nt-free, ATPγS or ADP states (71,72). These contrasting results show the need for further systematic studies on other helicases which could identify the relevant factors that enable or prevent a helicase to unfold a GQ in the absence of ATP. The answers to these questions could potentially challenge our current understanding of the mechanism behind helicase-mediated GQ unfolding.

Another important point highlighted in this study is the influence of the overhang on BLM-mediated GQ unfolding. Various helicases have been shown to possess GQ unfolding activities and the efficiencies of these activities show large variations (41,48). Our study suggests that the observed differences in the GQ unfolding activities of various helicases may partially be due to their ssDNA binding affinities and directionality and the lengths of ssDNA segments that are available in the vicinity of GQ constructs, or other secondary structures, used in such studies.

We demonstrate that BLM-mediated intramolecular GQ unfolding does not require ATP, and can occur in any nucleotide state including the nt-free state. Hence, translocase or helicase activity of BLM is not required for unfolding intramolecular GQ that has a long enough overhang. A limited range of measurements also showed WRN to have a similar GQ unfolding activity. RecQ and RECQ5 were able to unfold GQ only when the ionic concentration was lowered significantly below the physiological conditions, which resulted in a weaker GQ structure. The GQ unfolding activity of these proteins in the absence of ATP are in contrast to prior bulk studies performed on intermolecular GQs which showed GQ unfolding only in the presence of ATP. We also observe that BLM-mediated GQ unfolding is most efficient in the ATPγS state followed by the nt-free state and least efficient in the ADP state. This order correlates with BLM binding stability to the overhang ssDNA in these nucleotide states. We also show that BLM-mediated GQ unfolding becomes more efficient as the overhang length is increased or as the GQ stability is reduced. Finally, we showed that BLM does not show a significant conformation dependence in unfolding GQ. Our study raises a number of questions that challenge our current understanding of helicase-GQ interactions and the mechanism behind the GQ unfolding that results from these interactions. In particular, binding of proteins in the vicinity of GQ, even if the protein is not a translocase or translocation does not take place for different reasons, results in significant destabilization of GQ, which could be a major mechanism for resolving these structures inside the cells.

## SUPPLEMENTARY DATA

Supplementary Data are available at NAR Online.

SUPPLEMENTARY DATA

## References

[1] German J. (1993). Bloom syndrome: a mendelian prototype of somatic mutational disease. Medicine (Baltimore).

[2] Karow J.K., Chakraverty R.K., Hickson I.D. (1997). The Bloom's syndrome gene product is a 3′-5′ DNA helicase. J. Biol. Chem..

[3] Chakraverty R.K., Hickson I.D. (1999). Defending genome integrity during DNA replication: a proposed role for RecQ family helicases. Bioessays.

[4] Ellis N.A., Groden J., Ye T.Z., Straughen J., Lennon D.J., Ciocci S., Proytcheva M., German J. (1995). The Bloom's syndrome gene product is homologous to RecQ helicases. Cell.

[5] Kitao S., Ohsugi I., Ichikawa K., Goto M., Furuichi Y., Shimamoto A. (1998). Cloning of two new human helicase genes of the RecQ family: biological significance of multiple species in higher eukaryotes. Genomics.

[6] Vindigni A., Hickson I.D. (2009). RecQ helicases: multiple structures for multiple functions. HFSP J..

[7] Watt P.M., Hickson I.D. (1996). Failure to unwind causes cancer. Genome stability. Curr. Biol..

[8] Bachrati C.Z., Hickson I.D. (2003). RecQ helicases: suppressors of tumorigenesis and premature aging. Biochem. J..

[9] Opresko P.L., Cheng W.H., Bohr V.A. (2004). Junction of RecQ helicase biochemistry and human disease. J. Biol. Chem..

[10] Karow J.K., Newman R.H., Freemont P.S., Hickson I.D. (1999). Oligomeric ring structure of the Bloom's syndrome helicase. Curr. Biol..

[11] Xu Y.N., Bazeille N., Ding X.Y., Lu X.M., Wang P.Y., Bugnard E., Grondin V., Dou S.X., Xi X.G. (2012). Multimeric BLM is dissociated upon ATP hydrolysis and functions as monomers in resolving DNA structures. Nucleic Acids Res..

[12] Yodh J.G., Stevens B.C., Kanagaraj R., Janscak P., Ha T. (2009). BLM helicase measures DNA unwound before switching strands and hRPA promotes unwinding reinitiation. EMBO J..

[13] Barefield C., Karlseder J. (2012). The BLM helicase contributes to telomere maintenance through processing of late-replicating intermediate structures. Nucleic Acids Res..

[14] Hoadley K.A., Xu D., Xue Y., Satyshur K.A., Wang W., Keck J.L. (2010). Structure and cellular roles of the RMI core complex from the Bloom syndrome dissolvasome. Structure.

[15] Chaires J.B. (2010). Human telomeric G-quadruplex: thermodynamic and kinetic studies of telomeric quadruplex stability. FEBS J..

[16] Gray R.D., Chaires J.B. (2008). Kinetics and mechanism of K+- and Na+-induced folding of models of human telomeric DNA into G-quadruplex structures. Nucleic Acids Res..

[17] Lane A.N., Chaires J.B., Gray R.D., Trent J.O. (2008). Stability and kinetics of G-quadruplex structures. Nucleic Acids Res..

[18] Stegle O., Payet L., Mergny J.L., MacKay D.J., Leon J.H. (2009). Predicting and understanding the stability of G-quadruplexes. Bioinformatics.

[19] Blackburn E.H. (1991). Structure and function of telomeres. Nature.

[20] Sen D., Gilbert W. (1988). Formation of parallel four-stranded complexes by guanine-rich motifs in DNA and its implications for meiosis. Nature.

[21] Sundquist W.I., Klug A. (1989). Telomeric DNA dimerizes by formation of guanine tetrads between hairpin loops. Nature.

[22] Blackburn E.H., Greider C.W., Szostak J.W. (2006). Telomeres and telomerase: the path from maize, Tetrahymena and yeast to human cancer and aging. Nat. Med..

[23] Fletcher T.M., Sun D., Salazar M., Hurley L.H. (1998). Effect of DNA secondary structure on human telomerase activity. Biochemistry.

[24] Gellert M., Lipsett M.N., Davies D.R. (1962). Helix formation by guanylic acid. Proc. Natl. Acad. Sci. U.S.A..

[25] Gilbert D.E., Feigon J. (1999). Multistranded DNA structures. Curr. Opin. Struct. Biol..

[26] Williamson J.R. (1994). G-quartet structures in telomeric DNA. Annu. Rev. Biophys. Biomol. Struct..

[27] Eddy J., Maizels N. (2006). Gene function correlates with potential for G4 DNA formation in the human genome. Nucleic Acids Res..

[28] Eddy J., Maizels N. (2008). Conserved elements with potential to form polymorphic G-quadruplex structures in the first intron of human genes. Nucleic Acids Res..

[29] Huppert J.L., Balasubramanian S. (2005). Prevalence of quadruplexes in the human genome. Nucleic Acids Res..

[30] Huppert J.L., Balasubramanian S. (2007). G-quadruplexes in promoters throughout the human genome. Nucleic Acids Res..

[31] Todd A.K., Johnston M., Neidle S. (2005). Highly prevalent putative quadruplex sequence motifs in human DNA. Nucleic Acids Res..

[32] Du Z., Zhao Y., Li N. (2008). Genome-wide analysis reveals regulatory role of G4 DNA in gene transcription. Genome Res..

[33] Qin Y., Hurley L.H. (2008). Structures, folding patterns, and functions of intramolecular DNA G-quadruplexes found in eukaryotic promoter regions. Biochimie.

[34] Balasubramanian S., Hurley L.H., Neidle S. (2011). Targeting G-quadruplexes in gene promoters: a novel anticancer strategy. Nat. Rev. Drug Discov..

[35] Huppert J.L., Bugaut A., Kumari S., Balasubramanian S. (2008). G-quadruplexes: the beginning and end of UTRs. Nucleic Acids Res..

[36] Kumari S., Bugaut A., Huppert J.L., Balasubramanian S. (2007). An RNA G-quadruplex in the 5′ UTR of the NRAS proto-oncogene modulates translation. Nat. Chem. Biol..

[37] Morris M.J., Basu S. (2009). An unusually stable G-quadruplex within the 5′-UTR of the MT3 matrix metalloproteinase mRNA represses translation in eukaryotic cells. Biochemistry.

[38] Morris M.J., Negishi Y., Pazsint C., Schonhoft J.D., Basu S. (2010). An RNA G-quadruplex is essential for cap-independent translation initiation in human VEGF IRES. J. Am. Chem. Soc..

[39] Biffi G., Tannahill D., McCafferty J., Balasubramanian S. (2013). Quantitative visualization of DNA G-quadruplex structures in human cells. Nat. Chem..

[40] Lipps H.J., Rhodes D. (2009). G-quadruplex structures: in vivo evidence and function. Trends Cell. Biol..

[41] Paeschke K., Capra J.A., Zakian V.A. (2011). DNA replication through G-quadruplex motifs is promoted by the Saccharomyces cerevisiae Pif1 DNA helicase. Cell.

[42] Qureshi M.H., Ray S., Sewell A.L., Basu S., Balci H. (2012). Replication protein A unfolds G-quadruplex structures with varying degrees of efficiency. J. Phys. Chem. B..

[43] Ray S., Qureshi M.H., Malcolm D.W., Budhathoki J.B., Celik U., Balci H. (2013). RPA-mediated unfolding of systematically varying G-quadruplex structures. Biophys. J..

[44] Fernando H., Sewitz S., Darot J., Tavare S., Huppert J.L., Balasubramanian S. (2009). Genome-wide analysis of a G-quadruplex-specific single-chain antibody that regulates gene expression. Nucleic Acids Res..

[45] Ribeyre C., Lopes J., Boule J.B., Piazza A., Guedin A., Zakian V.A., Mergny J.L., Nicolas A. (2009). The yeast Pif1 helicase prevents genomic instability caused by G-quadruplex-forming CEB1 sequences in vivo. PLoS Genet..

[46] Sanders C.M. (2010). Human Pif1 helicase is a G-quadruplex DNA-binding protein with G-quadruplex DNA-unwinding activity. Biochem. J..

[47] Johnson J.E., Cao K., Ryvkin P., Wang L.S., Johnson F.B. (2010). Altered gene expression in the Werner and Bloom syndromes is associated with sequences having G-quadruplex forming potential. Nucleic Acids Res..

[48] Paeschke K., Bochman M.L., Garcia P.D., Cejka P., Friedman K.L., Kowalczykowski S.C., Zakian V.A. (2013). Pif1 family helicases suppress genome instability at G-quadruplex motifs. Nature.

[49] Hershman S.G., Chen Q., Lee J.Y., Kozak M.L., Yue P., Wang L.S., Johnson F.B. (2008). Genomic distribution and functional analyses of potential G-quadruplex-forming sequences in Saccharomyces cerevisiae. Nucleic Acids Res..

[50] Huber M.D., Lee D.C., Maizels N. (2002). G4 DNA unwinding by BLM and Sgs1p: substrate specificity and substrate-specific inhibition. Nucleic Acids Res..

[51] Mohaghegh P., Karow J.K., Brosh R.M., Bohr V.A., Hickson I.D. (2001). The Bloom's and Werner's syndrome proteins are DNA structure-specific helicases. Nucleic Acids Res..

[52] Sun H., Karow J.K., Hickson I.D., Maizels N. (1998). The Bloom's syndrome helicase unwinds G4 DNA. J. Biol. Chem..

[53] Kamath-Loeb A., Loeb L.A., Fry M. (2012). The Werner syndrome protein is distinguished from the Bloom syndrome protein by its capacity to tightly bind diverse DNA structures. PloS ONE.

[54] Huber M.D., Duquette M.L., Shiels J.C., Maizels N. (2006). A conserved G4 DNA binding domain in RecQ family helicases. J. Mol. Biol..

[55] Wang Q., Liu J.Q., Chen Z., Zheng K.W., Chen C.Y., Hao Y.H., Tan Z. (2011). G-quadruplex formation at the 3′ end of telomere DNA inhibits its extension by telomerase, polymerase and unwinding by helicase. Nucleic Acids Res..

[56] Liu J.Q., Chen C.Y., Xue Y., Hao Y.H., Tan Z. (2010). G-quadruplex hinders translocation of BLM helicase on DNA: a real-time fluorescence spectroscopic unwinding study and comparison with duplex substrates. J. Am. Chem. Soc..

[57] Janscak P., Garcia P.L., Hamburger F., Makuta Y., Shiraishi K., Imai Y., Ikeda H., Bickle T.A. (2003). Characterization and mutational analysis of the RecQ core of the Bloom syndrome protein. J. Mol. Biol..

[58] Bernstein D.A., Keck J.L. (2003). Domain mapping of Escherichia coli RecQ defines the roles of conserved N- and C-terminal regions in the RecQ family. Nucleic Acids Res..

[59] Garcia P.L., Liu Y., Jiricny J., West S.C., Janscak P. (2004). Human RECQ5beta, a protein with DNA helicase and strand-annealing activities in a single polypeptide. EMBO J..

[60] Orren D.K., Brosh R.M., Nehlin J.O., Machwe A., Gray M.D., Bohr V.A. (1999). Enzymatic and DNA binding properties of purified WRN protein: high affinity binding to single-stranded DNA but not to DNA damage induced by 4NQO. Nucleic Acids Res..

[61] Ambrus A., Chen D., Dai J., Bialis T., Jones R.A., Yang D. (2006). Human telomeric sequence forms a hybrid-type intramolecular G-quadruplex structure with mixed parallel/antiparallel strands in potassium solution. Nucleic Acids Res..

[62] Neff N.F., Ellis N.A., Ye T.Z., Noonan J., Huang K., Sanz M., Proytcheva M. (1999). The DNA helicase activity of BLM is necessary for the correction of the genomic instability of Bloom syndrome cells. Mol. Biol. Cell.

[63] Yang Y., Dou S.X., Xu Y.N., Bazeille N., Wang P.Y., Rigolet P., Xu H.Q., Xi X.G. (2010). Kinetic mechanism of DNA unwinding by the BLM helicase core and molecular basis for its low processivity. Biochemistry.

[64] Gyimesi M., Sarlos K., Kovacs M. (2010). Processive translocation mechanism of the human Bloom's syndrome helicase along single-stranded DNA. Nucleic Acids Res..

[65] Zhou R., Zhang J., Bochman M.L., Zakian V.A., Ha T. (2014). Periodic DNA patrolling underlies diverse functions of Pif1 on R-loops and G-rich DNA. eLife.

[66] Wlodarczyk A., Grzybowski P., Patkowski A., Dobek A. (2005). Effect of ions on the polymorphism, effective charge, and stability of human telomeric DNA. Photon correlation spectroscopy and circular dichroism studies. J. Phys. Chem. B.

[67] Guedin A., Gros J., Alberti P., Mergny J.L. (2010). How long is too long? Effects of loop size on G-quadruplex stability. Nucleic Acids Res..

[68] Tippana R., Xiao W., Myong S. (2014). G-quadruplex conformation and dynamics are determined by loop length and sequence. Nucleic Acids Res..

[69] Tran P.L., Mergny J.L., Alberti P. (2011). Stability of telomeric G-quadruplexes. Nucleic Acids Res..

[70] Wang Q., Ma L., Hao Y.H., Tan Z. (2010). Folding equilibrium constants of telomere G-quadruplexes in free state or associated with proteins determined by isothermal differential hybridization. Anal. Chem..

[71] Bharti S.K., Sommers J.A., George F., Kuper J., Hamon F., Shin-Ya K., Teulade-Fichou M.P., Kisker C., Brosh R.M. (2013). Specialization among iron-sulfur cluster helicases to resolve G-quadruplex DNA structures that threaten genomic stability. J. Biol. Chem..

[72] Gray L.T., Vallur A.C., Eddy J., Maizels N. (2014). G quadruplexes are genomewide targets of transcriptional helicases XPB and XPD. Nat. Chem. Biol..

