# Supplementary material for: RecQ-core of BLM unfolds telomeric G-quadruplex in the absence of ATP
Source: Nucleic Acids Res. 2014 Sep 22;42(18):11528–45. doi: 10.1093/nar/gku856 (PMC4191421; doi:10.1093/nar/gku856)
Supplement: SUPPLEMENTARY DATA [file supp_42_18_11528__index.html]

RecQ-core of BLM unfolds telomeric G-quadruplex in the absence of ATP — RecQ-core of BLM unfolds telomeric G-quadruplex in the absence of ATP — SUPPLEMENTARY DATA 

# RecQ-core of BLM unfolds telomeric G-quadruplex in the absence of ATP

## SUPPLEMENTARY DATA

**Files in this Data Supplement:**

- SUPPLEMENTARY DATA
