# Supplementary material for: RecQ-core of BLM unfolds telomeric G-quadruplex in the absence of ATP
Source: Nucleic Acids Res. 2014 Sep 22;42(18):11528–45. doi: 10.1093/nar/gku856 (PMC4191421; doi:10.1093/nar/gku856)
Supplement: SUPPLEMENTARY DATA [file supp_gku856_nar-02220-f-2014-File011.pdf]

Supplementary Data

BLM-mediated Telomeric G-quadruplex Unfolding in the Absence of ATP

Jagat B. Budhathoki[1], Sujay Ray[1], Vaclav Urban[2], Pavel Janscak[2,3], Jaya G. Yodh[4,*], Hamza Balci[1,*]

[1] Department of Physics, Kent State University, Kent, OH, 44242, USA

[2] Institute of Molecular Genetics AS CR, Prague, Czech Republic

[3] Institute of Molecular Cancer Research, University of Zurich, Zurich, Switzerland;

[4] Department of Physics and Center for the Physics of Living Cells, University of Illinois at Urbana-Champaign, Urbana, IL, 61801, USA

* To whom correspondence should be addressed. Tel: +1 (330) 672 2577; Fax: +1 (330) 672 2959; Email: hbalci@kent.edu

Correspondence may also be addressed to Jaya G. Yodh. Tel: +1 (217) 244 1155; Fax: +1 (217) 244 1155; Email: jyodh@illinois.edu

Identification of the Unfolded and BLM-bound Unfolded FRET States

The telomeric sequence we studied forms a stable GQ structure at 150 mM K^+. However, various other FRET states emerge upon introduction of BLM. Identifying the DNA conformations these new FRET states represent is complicated on a GQ forming sequence as the sequence could potentially form various secondary structures. In particular, we aimed to identify the FRET states representing the unfolded and BLM-bound unfolded states as these states would be expected to form upon interaction with BLM. To avoid such complications we performed smFRET measurements on a 35 nucleotide (nt) long polythymine DNA construct (pd-polyT35, see Table 1 in manuscript for sequence), which is hybridized with the RNA-Stem to form a pdDNA construct similar to that used for GQ forming sequences. The single strand DNA (ssDNA) section of this construct has the same length as that of pd-hGQ12T, i.e. they are both 35 nt long. Therefore, it can be used as a model to represent the unfolded and BLM-bound unfolded states of pd-hGQ12T. Figure S1-A shows the smFRET distribution at 150 mM K^+ in the absence of BLM. The peak at E_{FRET} = 0.40 corresponds to the coiled DNA structure for a 35 nt long ssDNA, which is equivalent to the unfolded structure of pd-hGQ12T. Figure S1-B shows the smFRET distribution when 50 nM BLM is introduced to the sample chamber. In addition to the peak at E_{FRET} = 0.40, a new peak is observed at E_{FRET} = 0.20 which we interpret as the BLM-bound peak. Therefore, E_{FRET} =0.20 represents the BLM-bound unfolded peak of pd-hGQ12T.

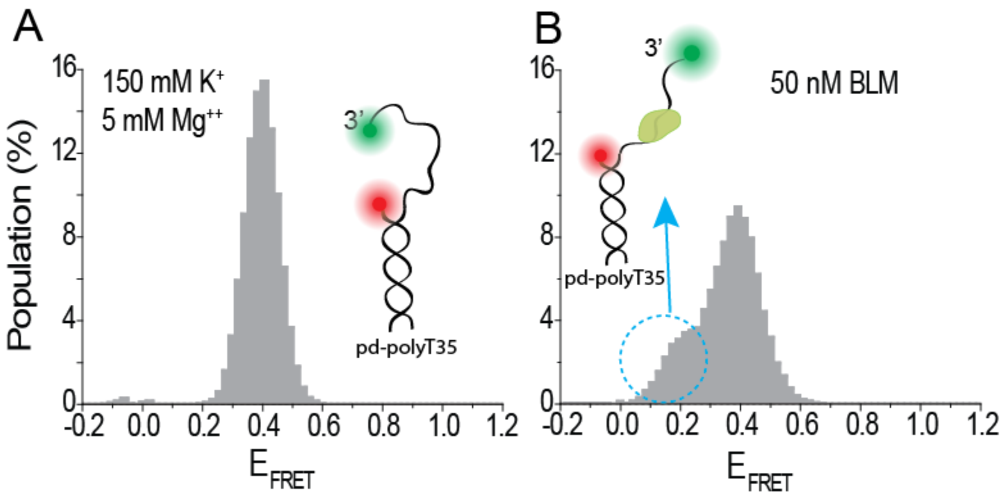

Supplementary Figure S1: smFRET measurements on pd-polyT35 construct which has a 35 nt long polythymine ssDNA tail, which is same length as the ssDNA section of pd-hGQ12T. pd-polyT35 is used to characterize the FRET levels corresponding to the unfolded and BLM-bound unfolded states of pd-hGQ12T. (A) smFRET distribution at 150 mM K^+ before BLM is added. The peak at E_{FRET} = 0.40 corresponds to the coiled DNA structure for a 35 nt long ssDNA, which is equivalent to the unfolded structure of pd-hGQ12T. The inset shows a schematic of the DNA construct. (B) smFRET distribution after adding 50 nM BLM to the chamber. The new peak at E_{FRET} = 0.20 represents the BLM-bound peak, which corresponds to the BLM-bound unfolded peak of pd-hGQ12T. The inset shows a schematic of the BLM-DNA complex represented by the peak at E_{FRET} = 0.20.

Circular Dichroism Assay and smFRET Data on Internally Labeled DNA Construct

Circular dichroism measurements were performed on pd-hGQ12T to obtain an independent confirmation of BLM-mediated GQ unfolding in the absence of ATP. The DNA concentration needs to be maintained at 1 μM to achieve a reliable CD signal. Therefore, we performed the measurements only at the highest protein concentration possible in our system -1 μM- in order to attain a significant change in the CD spectrum. Figure S2-A shows these data before and after adding 1 μM BLM to a cuvette that contains folded pd-hGQ12T in 150 mM K^+. The peak at ~290 nm is a characteristic signature of GQ structure, which diminishes upon addition of 1 μM BLM, which is consistent with GQ unfolding by BLM. Another smFRET DNA construct was designed in order to more directly probe GQ unfolding in which the donor fluorophore is moved from the 3' end to an internal site a couple nucleotides away from the GQ (inset of Figure S2-B).). This construct, pd-hGQ-2T-Cy3-10T, was formed by hybridizing the 18 nt RNA-Stem with 5'- TGG CGA CGG CAG CGA GGC TTG GGT TAG GGT TAG GGT TAG GG TT-Cy3-10T. pd-hGQ-2T-Cy3-10T eliminates any significant FRET changes that might take place due to binding of the protein to the overhang, and results in a significant FRET change only when the GQ is unfolded. The measurements on this construct resulted in considerable reduction in BLM-mediated GQ unfolding at 150 mM K^+, which is considered to be due to interference caused by the donor fluorophore on BLM-GQ interactions (data not shown). Nevertheless, a clear BLM-mediated unfolded GQ population was observed in both nt-free and 1 mM ATPγS states in 1 μM BLM. Figure S2-B and S2-C show these data in 50 mM K^+ and 10 mM K^+, respectively. Figure S2-D shows the quantification of the unfolded GQ population for the data presented in Figure S2-B and S2-C.

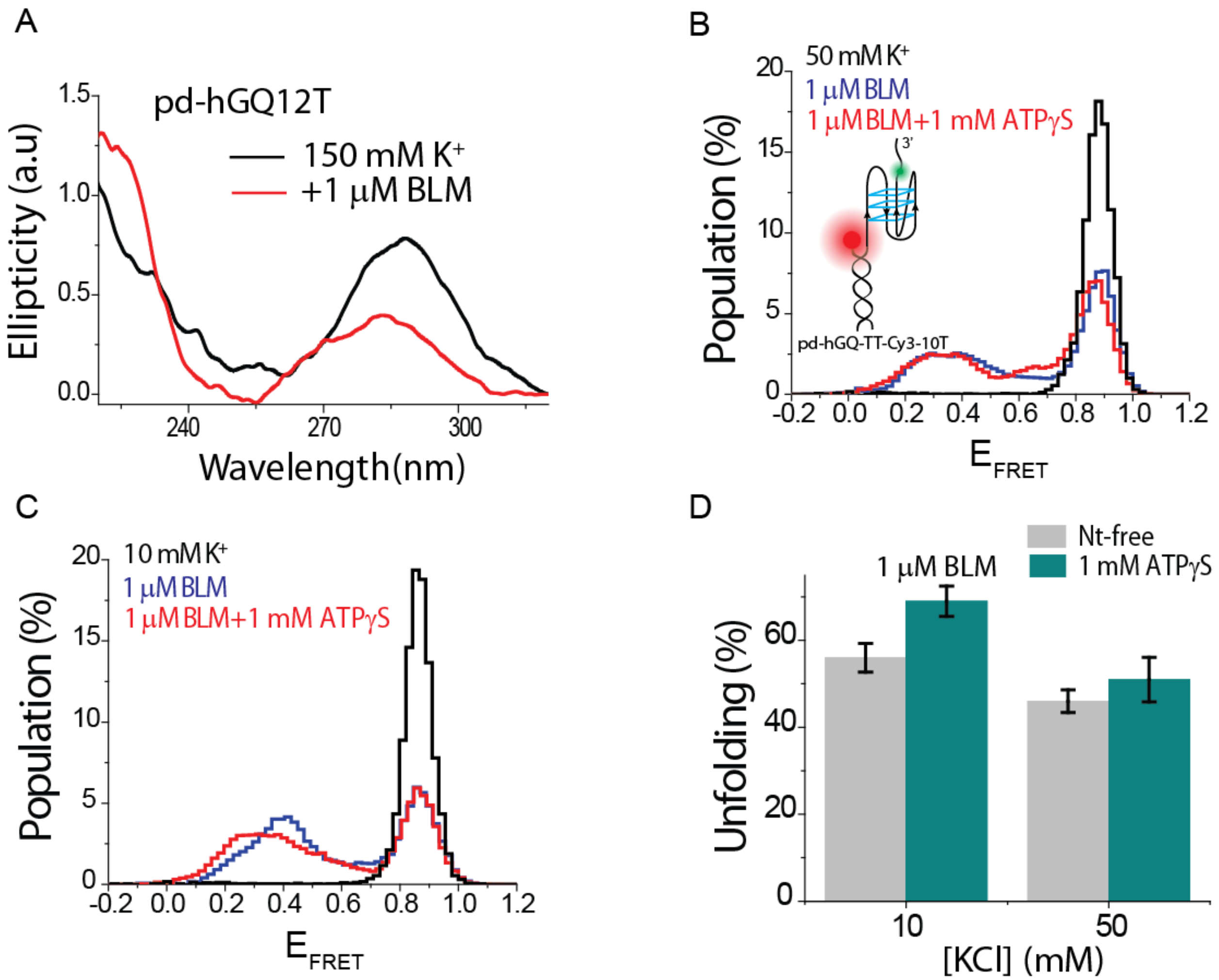

Figure S2: (A) CD data on pd-hGQ12T with and without 1 μM BLM. **(B)** and **(C)** smFRET data on pd-hGQ-TT-Cy3-10T in 50 mM and 10 mM K^+, respectively. **(D)** Quantitation of the unfolded GQ population in the histograms in (B) and (C).

FRET Histograms and Subtraction Analysis for BLM Titration on pd-hGQ12T

Figure 1 of the manuscript shows sample histograms of BLM-GQ interactions when BLM is titrated under different nucleotide conditions for the pd-hGQ12T construct. Figure S3 is an extension of this figure that shows the histograms for the BLM concentrations not shown in Figure 1. Figure S4 shows the corresponding histograms of subtraction analysis we performed to quantify BLM-mediated GQ unfolding. For all the subtraction analysis presented in Figure S4, the folded state before BLM is introduced to chamber is taken as the reference. This folded state does not vary upon introduction of ATPγS or ADP to chamber as the ionic strength - which determines GQ stability -is kept constant. In Figure S3 and S4, the nucleotide states and the BLM concentrations are written on the graphs. All experiments were performed at 150 mM K^+.

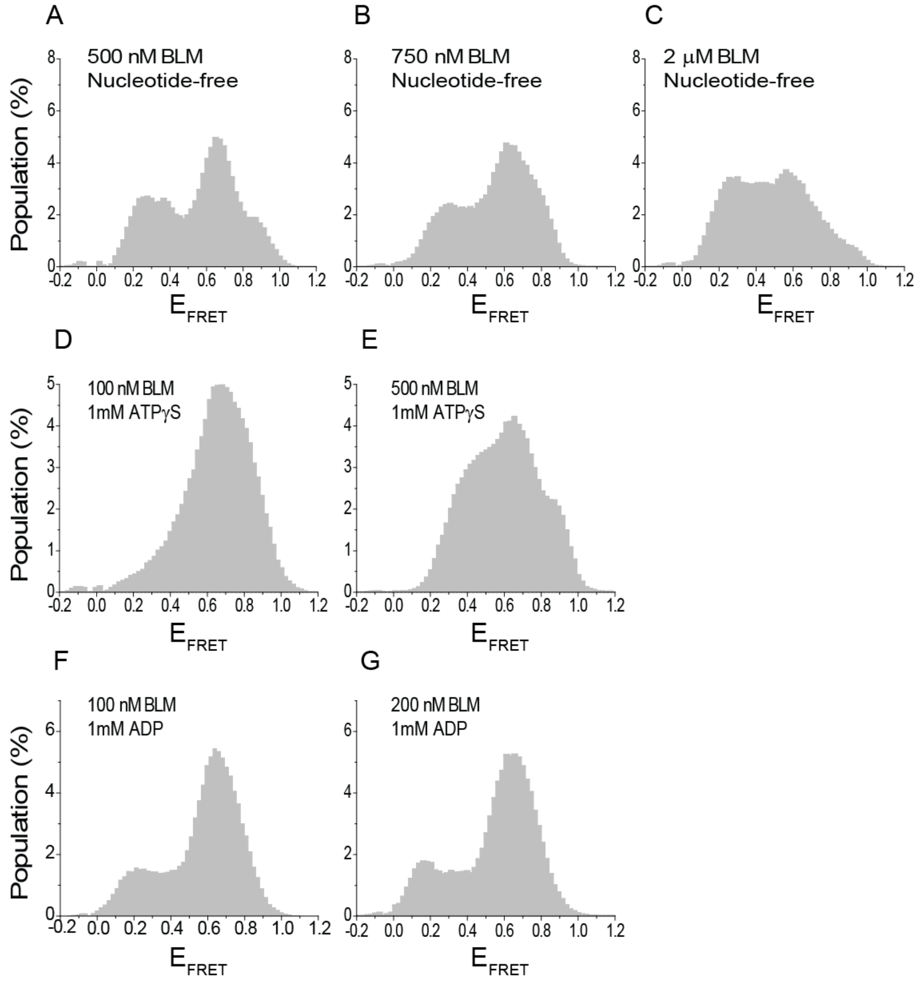

Supplementary Figure S3: smFRET histograms showing the BLM concentrations we studied, other than those shown in Figure 1 of the manuscript, in the nucleotide-free, 1 mM ATPγS, or 1 mM ADP states. The data shown in **(A)-(C)** were taken in the nucleotide-free state at 500 nM, 750 nM, and 1 μM BLM concentration, respectively. The data shown in **(D)-(E)** were taken at 1 mM ATPγS at 100 nM and 500 nM BLM concentration, respectively. The data shown in **(F)-(G)** were taken at 1 mM ADP at 100 nM and 200 nM BLM concentration, respectively. The BLM concentration and the nucleotide state for each histogram are written on the graphs.

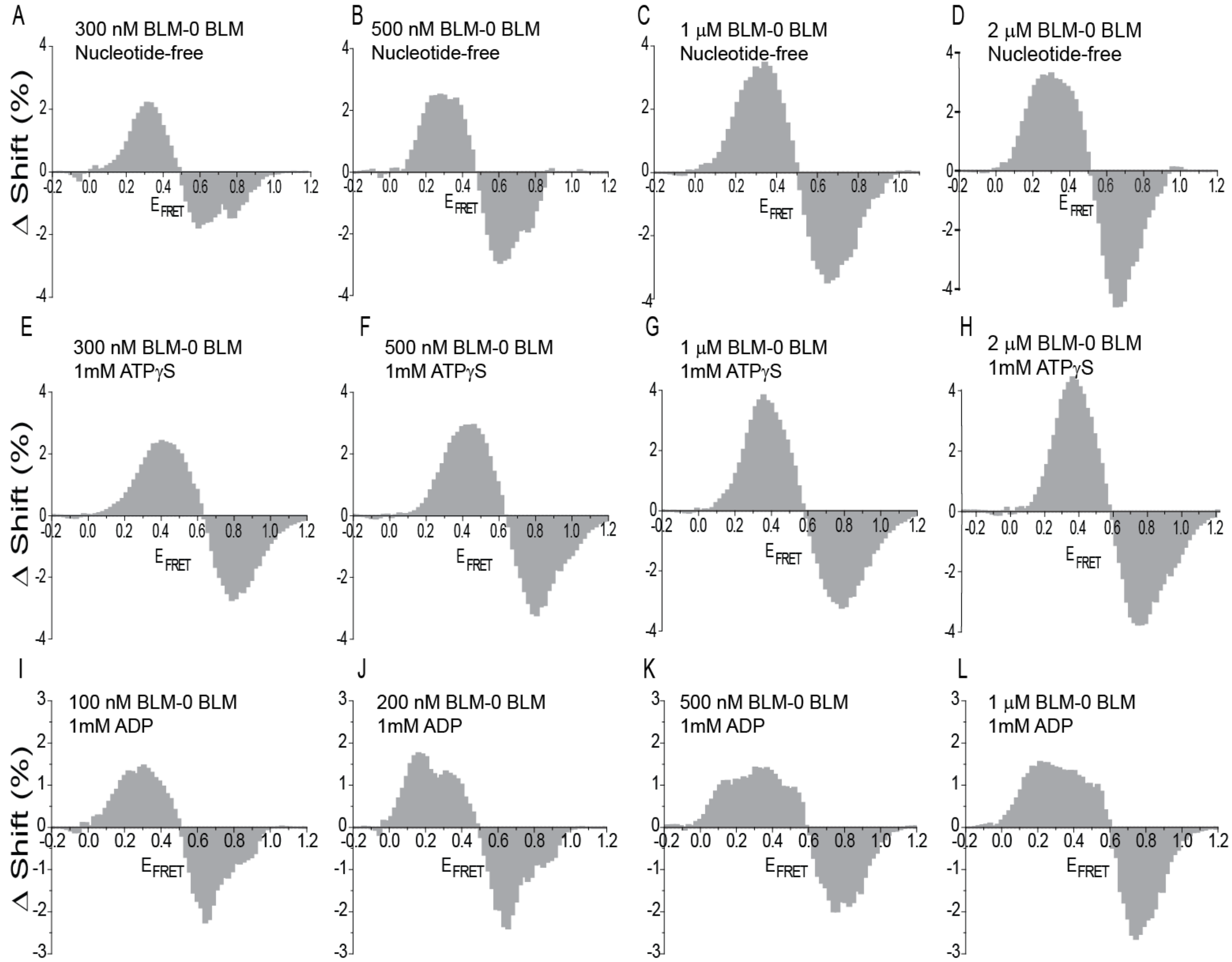

Supplementary Figure S4: Subtraction analysis performed on the data shown in Figure 1 and Figure S3 using the folded state shown in Figure 1B as the reference. This folded state is the same for all nucleotide-states in the absence of BLM, enabling its use as the reference state for all the presented data in Figure 1 and Figure S3. The subtraction analysis was performed for data taken in **(A)-(D)** Nucleotide-free state; **(E)-(H)** 1 mM ATPγS; and **(I)-(L)** 1 mM ADP. The BLM and nucleotide concentrations used for each histogram are written on the graphs. These data were included in the Langmiur isotherms shown in Figure 1.

Electrophoretic Mobility Shift Assay of BLM binding to GQ constructs

A native PAGE gel binding assay of BLM binding to pd-hGQ12T GQ construct is shown in Figure S8. This assay is not capable of distinguishing between BLM-bound folded and BLM-bound unfolded GQ as only a single shifted band is observed upon introducing BLM. Therefore, this observation only confirms binding of BLM to the DNA substrate. The measurements were performed at 0, 50, 100, 150, 200, 300, 400, 500, and 1000 nM BLM concentrations, while the DNA concentration was 5 nM. The buffer contained 50 mM K^+, 5 mM Mg^{++}, 50mM Tris-HCl (pH7.5), and 1 mM DTT. The constructs were annealed via heating/slow cooling prior to the gel binding assay. BLM-DNA binding reactions were carried out at 22 °C for 10 minutes, followed by electrophoresis through a 4-20% native PAGE gel at 4 °C. The gel was run at 50 V for 5-8 hours in 0.5xTBE buffer. Figure S5-A shows the gel image and Figure S5-B shows a quantification of the unfolded band. The data were fit by Hill equation, which results in K_A=139 nM BLM which is ~5x greater than that observed in smFRET measurements performed in 25 °C. We attribute this difference to lower temperature of the EMSA measurements in which BLM activity is lower and GQ is more stable.

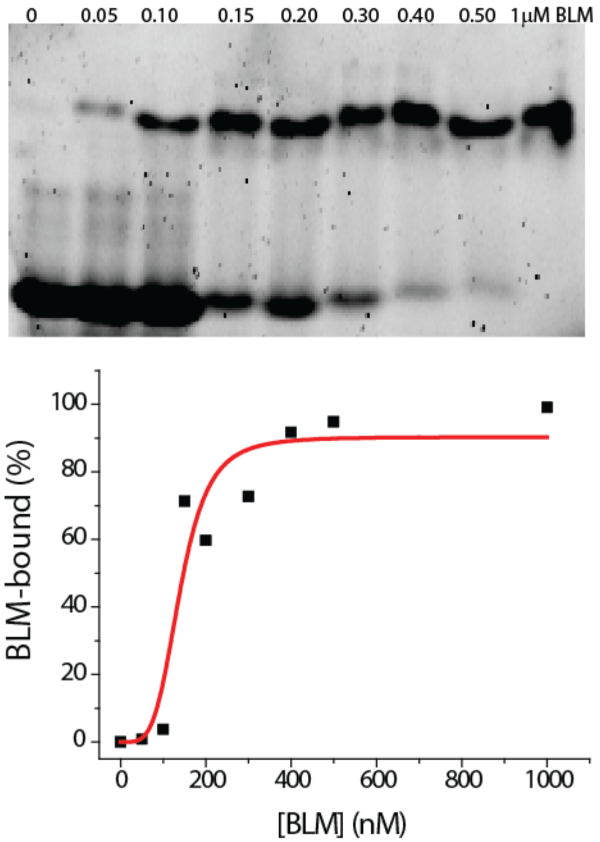

Supplementary Figure S5: Electrophoretic Mobility Shift Assay of BLM binding to pd-hGQ12T. Gels were imaged using a Molecular Dynamics Typhoon 9400 Multilaser Scanner. Cy5 image is shown. The lower panel shows quantification of the shifted band, for which we used GelQuant.NET software provided by biochemlabsolutions.com.

Measurements with Denatured Proteins and Control Measurements on Storage Buffer

Due to the unexpected nature of the observation that BLM unfolds GQ in the absence of ATP, we sought to ensure that the reason for GQ destabilization is not related to the buffer that BLM is stored in (50 mM Tris-HCl (pH=7.5), 200 mM NaCl, 50 % (v/v) glycerol and 1 mM DTT). The BLM is significantly diluted in imaging buffer, which contains 150 mM K^+ and 5 mM Mg^{++}, before it is added to the sample chamber. Therefore, any GQ destabilizing agent (such as a metal chelator) present in the storage buffer would be at a significantly less concentration. Nevertheless, we performed control measurements to study the influence of the storage buffer on the folded GQ state and did not observe any destabilization induced by this buffer in the absence of BLM. Figure S6 shows these data.

Another control measurement we performed was to denature BLM by heating it at 95 °C for 5 minutes before adding it to the sample chamber that contains folded GQ molecules. This measurement was an attempt to ensure that GQ destabilization is due to native BLM protein. We did not observe any GQ unfolding with 1 μM denatured BLM in either nt-free state or in 1 mM ATPγS state, as shown in Figure S6.

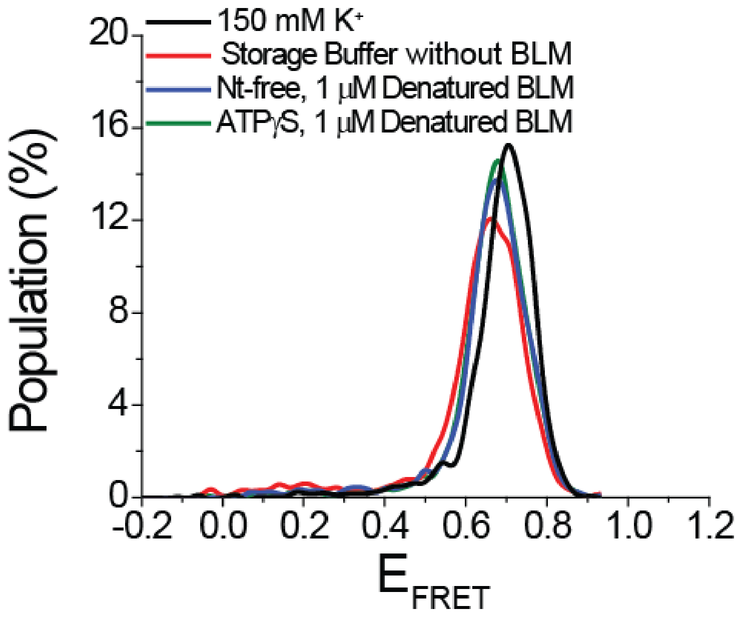

Supplementary Figure S6: Control measurements testing the storage buffer and thermally denatured BLM. GQ is not destabilized by any agent that might be in the storage buffer in the absence of BLM. Also, thermally denatured BLM does not destabilize GQ in either nt-free or ATPγS states.

Measurement to Test for Possible ATP Contamination of Purified BLM

In order to ensure that our smFRET assays probing BLM-mediated GQ unfolding do not contain ATP that is not accounted for, possibly ATP that might co-purify with BLM, we performed duplex DNA unwinding assays without adding any ATP to the medium. We used a forked DNA construct that was used by Yodh *et al.* (The EMBO Journal (2009) 28, 405–416), as shown in Figure S7-A. Unwinding of this duplex requires translocation along the ssDNA tracking strand in the 3'-5' direction. Figure S7-B shows that even saturating concentrations of BLM (1 μM) do not unwind the dsDNA in the nt-free state or in the presence of 1 μM ATPγS. On the other hand 10 nM BLM gives rise to visible dsDNA unwinding in the presence of 100 μM ATP. Figure S7-B shows an example trace showing such a repetitive unwinding event in agreement with those observed by Yodh *et al.* (The EMBO Journal (2009) 28, 405–416). The bottom panel in Figure S7-C is a zoomed in version of Figure S7-B to better show the repetitive unwinding events.

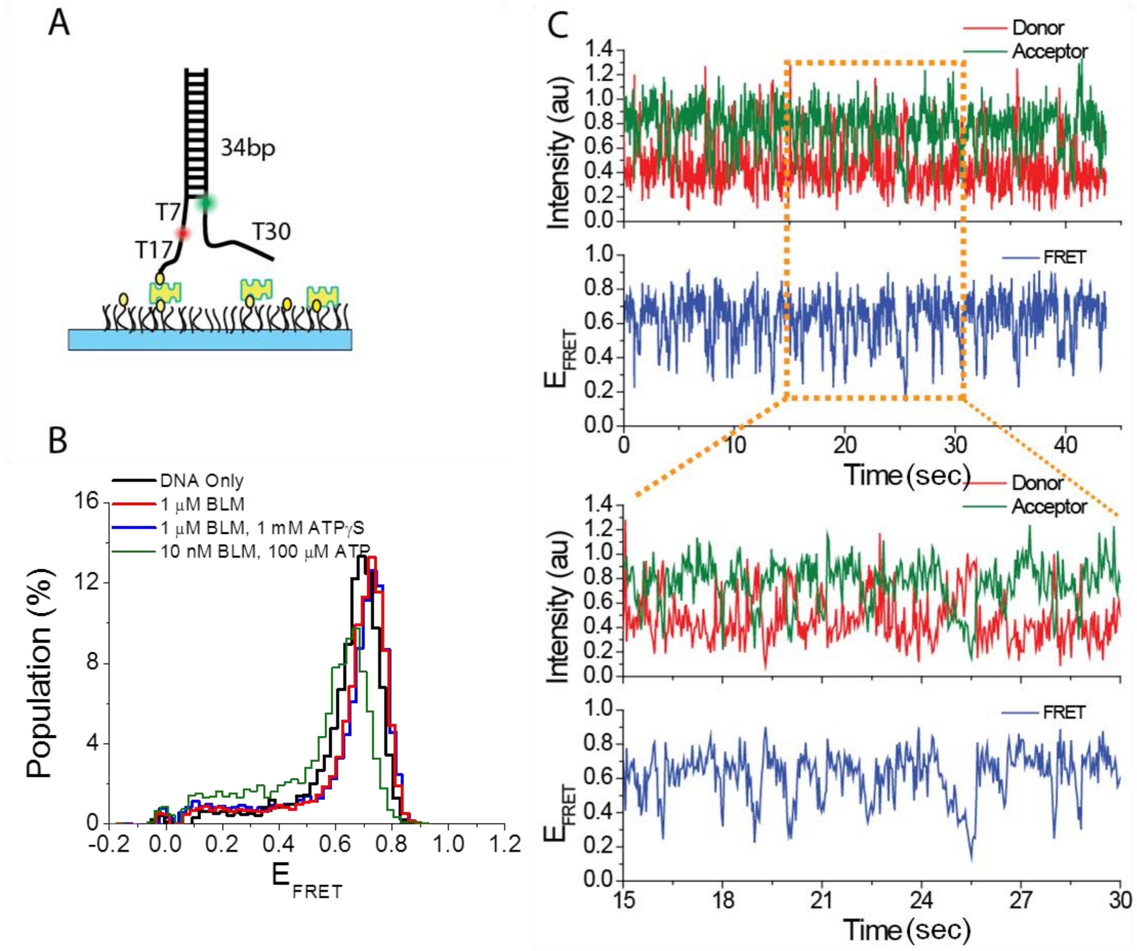

Supplementary Figure S7: Control measurements performed to ensure that our assays are not contaminated by ATP that co-purifies with BLM. **(A)** The forked DNA unwinding construct used in these studies. **(B)** BLM does not unwind dsDNA in the absence of ATP. **(C)** BLM catalyzes repetitive unwinding events only in the presence of ATP. The bottom panel is a zoomed in version of the top panel in the time interval between 15-30 seconds.

SDS-PAGE Measurements of Purified BLM

The purity of the BLM used in the single molecule FRET measurements was analyzed by 12% SDS-PAGE. Figure S8, displays a coomassie-stained 12% SDS-PAGE of core-BLM (1-5 μg) from which we estimate purity at >98%. Based on this, it is highly unlikely that BLM used in this study is contaminated with other proteins which are known to destabilize GQ in the absence of ATP, such as ssDNA binding proteins.

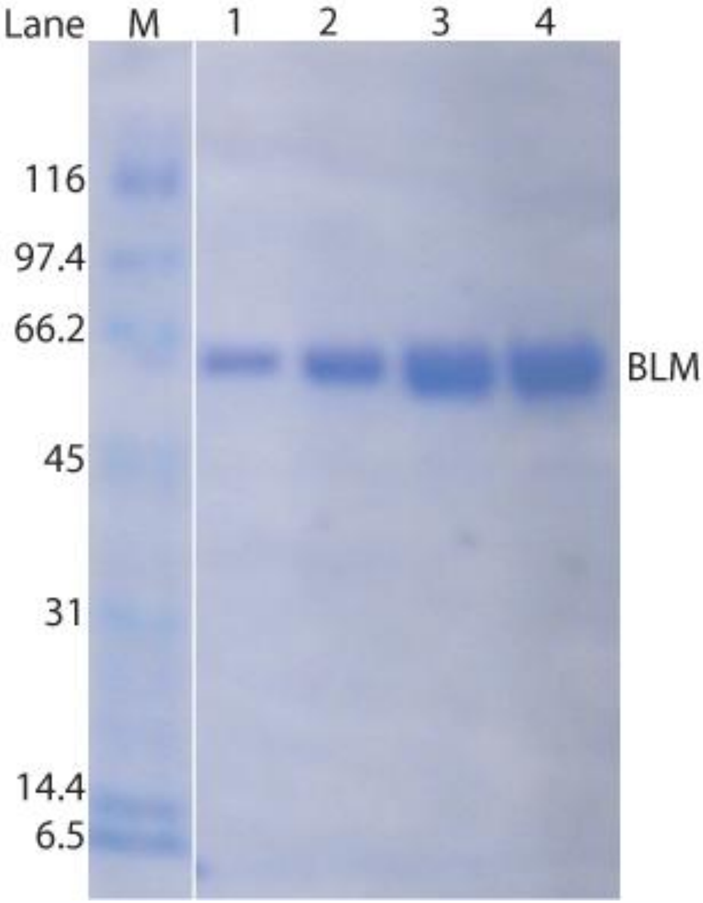

Supplementary Figure S8: Coomassie-stained 12% SDS-PAGE gel of purified Core BLM for assessment of BLM purity. Lane M includes protein size standards with the indicated molecular weight (kD). Lanes 1, 2, 3, and 4 contain 1, 2, 4, and 5 μg purified core BLM, respectively. A single band (estimate > 98% pure) is observed at the expected molecular weight for BLM. Thus , the probability is very low that another protein, such as an ssDNA binding protein that might be capable of unfolding GQ in the absence of ATP, copurifies with BLM.

GQ Unfolding by a BLM Mutant that does not Hydrolyze ATP

In order to directly demonstrate that helicase activity is not required for BLM-mediated GQ unfolding, a BLM mutant which does not hydrolyze ATP was generated by mutating the lysine at aminoacid 695 of $BLM^{(642\text{-}1290)}$ with methionine. This construct, BLM^{K695M}, was purified following a similar protocol to that of core BLM. The final BLM^{K695M} stock had a concentration of 1.78 μM and was in a buffer containing 400 mM Na^+. As the protein stock was in Na^+, we performed the smFRET measurements in 150 mM Na^+. In these measurements, the total Na^+ concentration was maintained at 150 mM by adjusting the additional Na^+ added to the sample chamber taking into account the salt that is introduced from the protein stock.

In order to ensure BLM^{K695M} does not have helicase activity, we introduced ATP and BLM^{K695M} to chamber that has surface immobilized pd-hGQ12T, which has a 3' tail. Under these circumstances, an active helicase would unfold the GQ and unwind the duplex stem, releasing the Cy3 strand. The Cy5 strand will continue to remain on the surface as it is bound via a biotin-neutravidin linker. We monitored the number of molecules that had Cy3 or Cy5 before and after adding 250 nM BLM^{K695M} and 1 mM ATP to the chamber. The Cy5 molecules were directly imaged by a red (632.8 nM) excitation laser, while green (532 nm) excitation illuminates all Cy3 and some Cy5 molecules (those that are within FRET range of an active Cy3 molecule). We did not observe any significant change in the number Cy3 molecules upon addition of BLM^{K695M} and ATP, which demonstrates that the duplex was not unwound by BLM^{K695M} (Figure S9). On the other hand, adding 250 nM core-BLM and 1 mM ATP to the same chamber nearly eliminated all Cy3 molecules while no significant change was observed in the number of Cy5 molecules, clearly demonstrating helicase activity.

Figure S10A-E show interactions of BLM^{K695M} with GQ in the nt-free state. The folded state in the absence of protein has a single high FRET peak (Figure S10-A), and introducing BLM^{K695M} at different concentrations results in a lower FRET peak representing the unfolded GQ population (Figure S10B-D). As BLM^{K695M} cannot hydrolyze ATP, even if there is ATP contamination in the protein stock it should not be hydrolyzed by BLM^{K695M}. Figure S10-E show the unfolded pd-hGQ12T population as a function of BLM^{K695M} concentration, and the red line is Langmuir isotherm fit to the data which results in α=61% and K_{eq}=133 nM. The unfolding activity of BLM^{K695M} is similar to that of the core-BLM, demonstrating that helicase activity is not required for protein-mediated GQ unfolding.

In order to ensure that this activity is also observed in the presence of K^+, we performed measurements in which the total ion concentration (Na^+ and K^+ in total) is kept at 150 mM. In these measurements, a certain concentration of Na^+ is introduced to the chamber by the protein stock, which was supplemented by the additional K^+ in the imaging buffer to reach a total monovalent ion concentration of 150 mM. For example, when 150 nM BLM^{K695M} is added to the chamber, 30 mM Na^+ is introduced from the protein stock. In order to reach 150 mM concentration, 120 mM K^+ is introduced by the imaging buffer. Similarly, in the case of 300 nM BLM^{K695M}, 60 mM Na^+ is introduced from the protein stock and 90 mM K^+ was added in the imaging buffer. Given that core-BLM shows similar GQ unfolding activity in 150 mM K^+ and 150 mM Na^+, the observed GQ unfolding in these cases should be similar to 150 mM Na^+ case. We tested 150 nM and 300 nM BLM^{K695M} in the ion concentrations stated above and obtained similar GQ unfolding to the 150 mM Na^+ case, as expected. These data are shown in Figure S10-F.

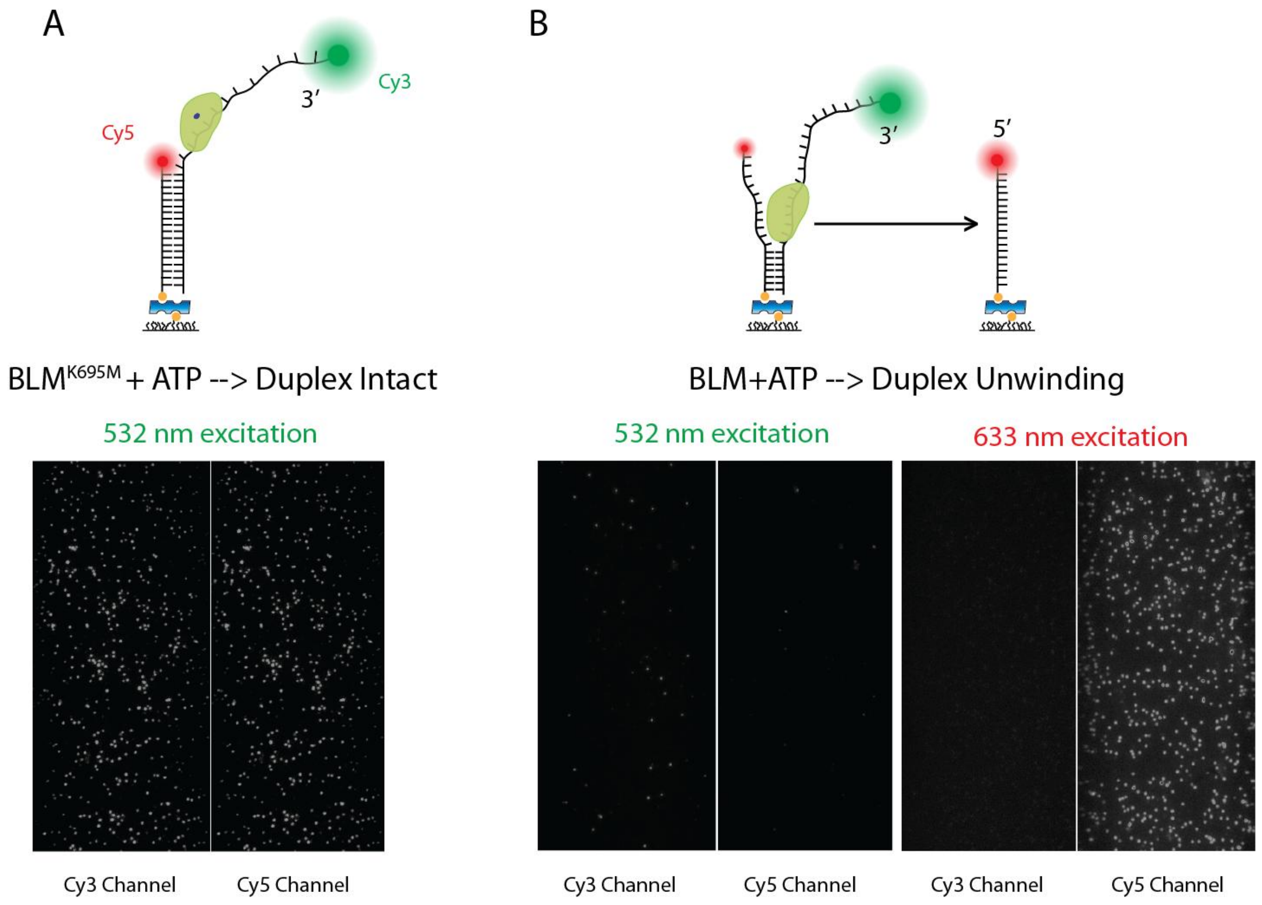

Supplementary Figure S9: smFRET measurements demonstrating that BLM^{K695M} does not unwind duplex DNA in the presence of ATP, while core-BLM unwinds the duplex DNA under the same conditions. Duplex unwinding is demonstrated by the release of the Cy3 strand from the surface, while the Cy5 strand remains bound.

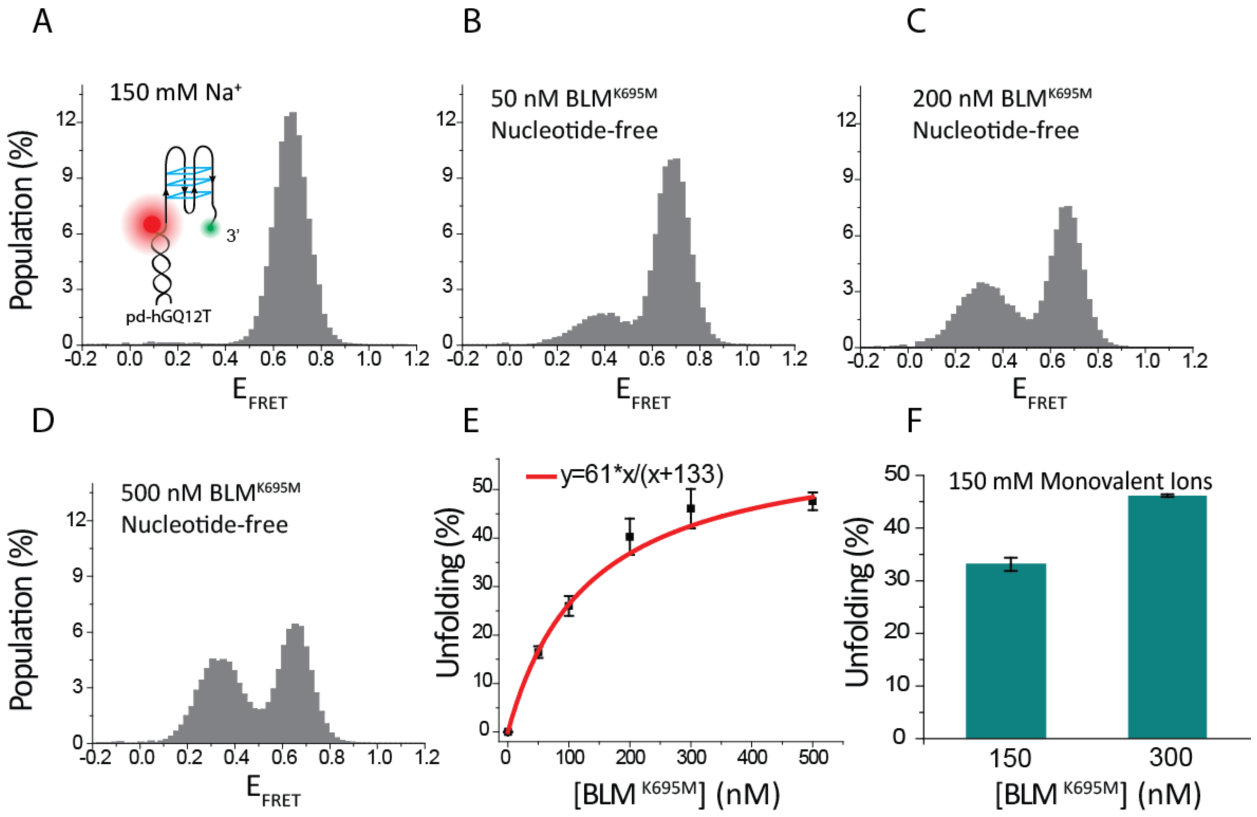

Supplementary Figure S10: smFRET measurements on pd-hGQ12T in 150 mM Na^+ at 0, 50, 200, and 500 nM BLM^{K695M} are shown in **(A)-(D)**, respectively. The data was taken in the nt-free state. **(E)** Langmuir binding isotherm fit was performed to quantify BLM^{K695M} mediated GQ unfolding activity in the nt-free state. **(F)** The unfolded pd-hGQ12T population is shown in the bar graph for 150 nM BLM^{K695M} (in 30 mM Na^+ and 120 mM K^+) and 300 nM BLM^{K695M}.

BLM-Mediated GQ Unfolding in AMP-PNP State

In order to confirm our results with another non-hydrolysable ATP analog, we performed BLM-mediated GQ interactions in the presence of AMP-PNP. We performed AMP-PNP titration measurements in the presence of 1 μM BLM and quantified the unfolded GQ population by performing subtraction analysis using the 1 μM BLM nt-free state as the reference. These data are shown in Figure S11. A Langmuir binding isotherm fit to the data results in α=18±1 % and K_{eq}=2.1±0.3 nM AMP-PNP. These results are consistent with the ATPγS, another non-hydrolysable ATP analog, measurements reported in Figure 2. Both ATPγS and AMP-PNP resulted in significantly more BLM-mediated GQ unfolding compared to the nt-free state.

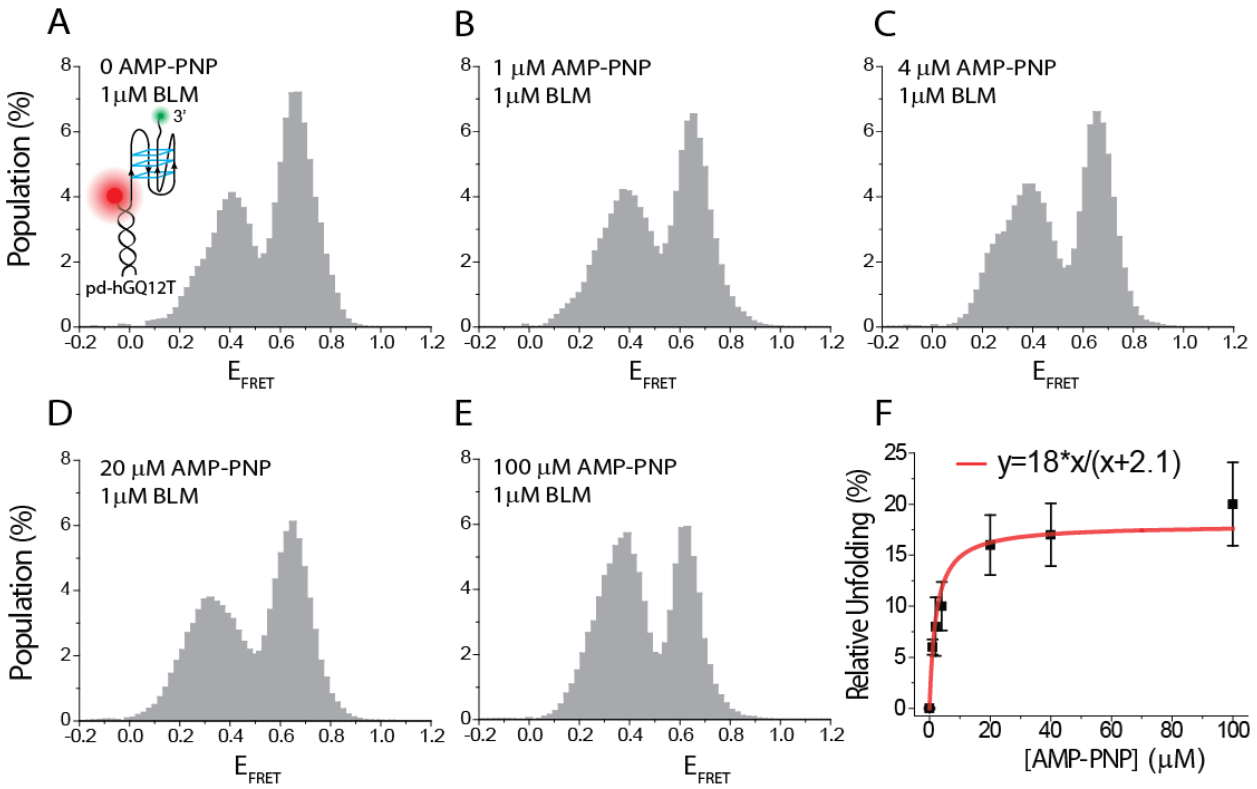

Supplementary Figure S11: smFRET measurements on pd-hGQ12T in 1 μM BLM in 0, 1 μM, 4 μM, 20 μM, and 100 μM AMP-PNP. Langmuir binding isotherm fit is shown in the bottom-right panel.

FRET Histograms and Subtraction Analysis for ATPγS and ADP Titration on pd-hGQ12T

Figure 2 of the manuscript shows sample histograms of BLM-GQ interactions when ATPγS or ADP concentration is titrated at 1 μM BLM concentration for the pd-hGQ12T construct. Figure S12 shows the histograms for the ATPγS and ADP concentrations that were not shown in Figure 2. Figure S13 shows the corresponding histograms of subtraction analysis which we used to quantify BLM-mediated GQ unfolding. The BLM and nucleotide concentration used for each histogram are written on the graph. All experiments were performed at 150 mM K^+. Data were included in the Langmuir isotherms shown in Figure 2.

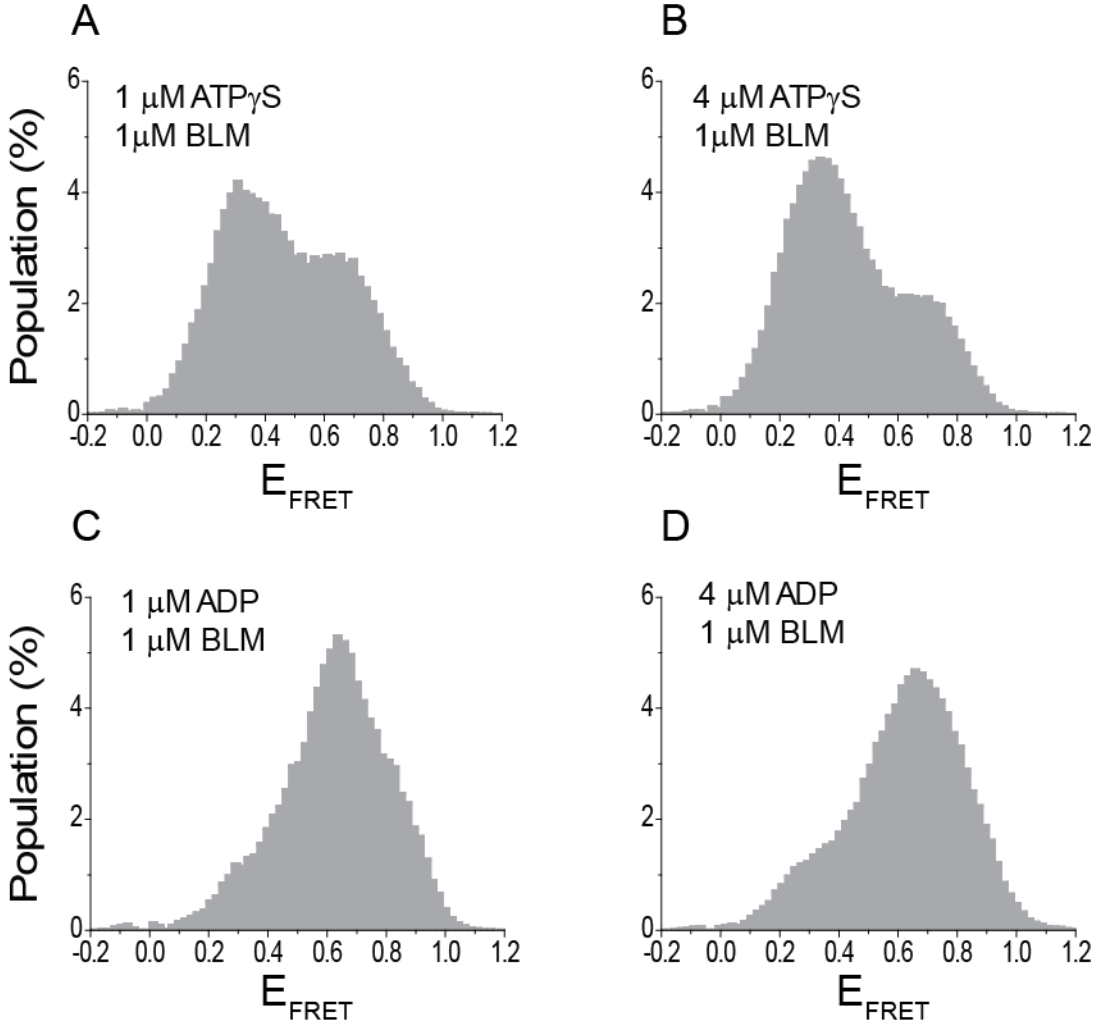

Supplementary Figure S12: smFRET histograms for ATPγS and ADP concentrations we studied, other than those shown in Figure 2 of the manuscript. All data are at 1 μM BLM concentration. **(A)** 1 μM ATPγS. **(B)** 4 μM ATPγS. **(C)** 1 μM ADP. **(D)** 4 μM ADP. The BLM and nucleotide concentrations used for each histogram is indicated on the graphs.

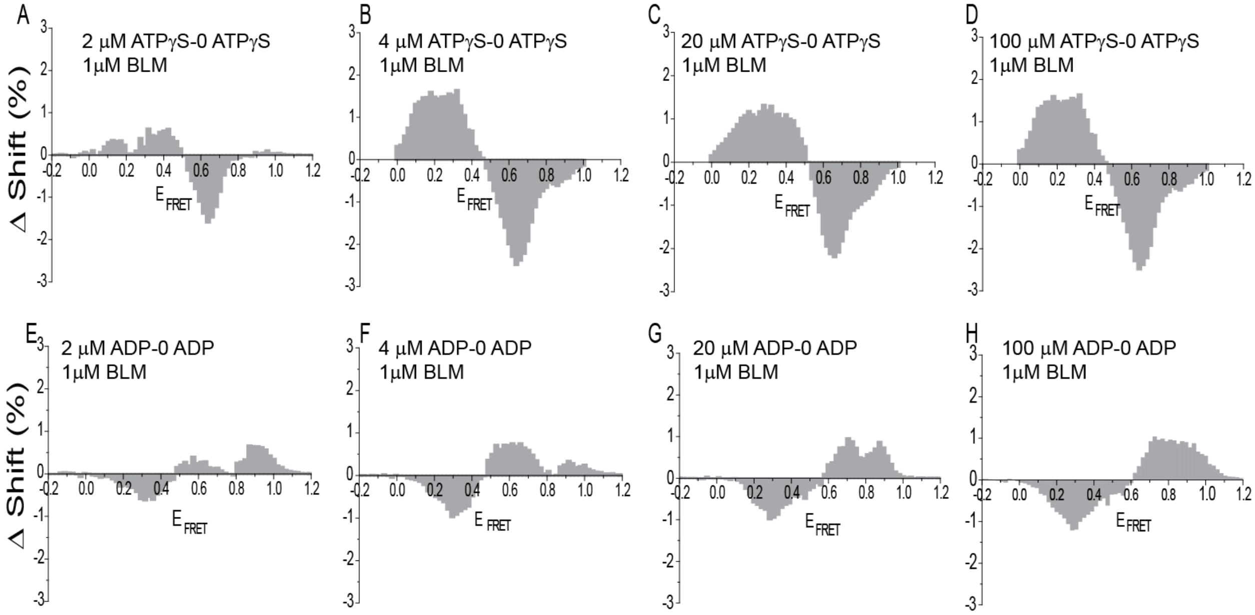

Supplementary Figure S13: Subtraction analysis performed on the data shown in Figure 2 and Figure S12 using the nt-free state at 1 μM BLM concentration as the reference state (Figure 1D of the manuscript). The subtraction analysis was performed for data taken at 2 μM, 4 μM, 20 μM, and 100 μM ATPγS concentrations as shown in **(A)-(D)**, respectively. Similarly, the subtraction analysis was performed for data taken at 2 μM, 4 μM, 20 μM, and 100 μM ADP concentration as shown in **(E)-(H)**, respectively. The BLM and nucleotide concentrations used for each histogram are indicated on the graphs.

smFRET Measurements on a GQ with a 15-nt long overhang

In order to ensure that the measurements on pd-hGQ12T are not compromised by the possibility of the ssDNA overhang being shorter than the BLM footprint, we repeated these measurements on pd-hGQ15T, which has an 15 nt long overhang. This 15 nt overhang is longer than the reported 14 nt footprint (Gyimesi *et al.* Nucleic Acids Research, 38, 4404-4414 (2010)), providing an opportunity for more stable binding of BLM to the overhang. The results of this study are shown in Figure S14. In this figure, we compared BLM-mediated GQ unfolding under different nucleotide-states, at saturating BLM and nucleotide concentrations. Figure S14A shows that the GQ stably folds at 150 mM K^+ and pH 7.5, even though GQ stability is known to decrease as the overhang gets longer (Hatzakis et al. Biochemistry, 49, 9152-9160 (2010)). Moreover, single-molecule time traces in the folding state were observed to be steady over the time of recording (~2 min.), suggesting that the folded GQ is robust even with this overhang. Figure S14B-D show the data in nt-free , 1 mM ATPγS, and 1 mM ADP states, respectively, which were all acquired at a constant BLM concentration of 1 μM. Figure S14-E shows a comparison of BLM-mediated GQ unfolding for different nucleotide states when the folded FRET distribution in Figure S14-A is subtracted from the data in these nucleotide states (similar to the analysis shown in Figure 1E). BLM-mediated GQ unfolding on pd-hGQ15T is most efficient in the ATPγS state (57% of GQ molecules unfolded), followed by nt-free state (48%), and is least efficient in the ADP state (22%), similar to the results with pd-hGQ12T.

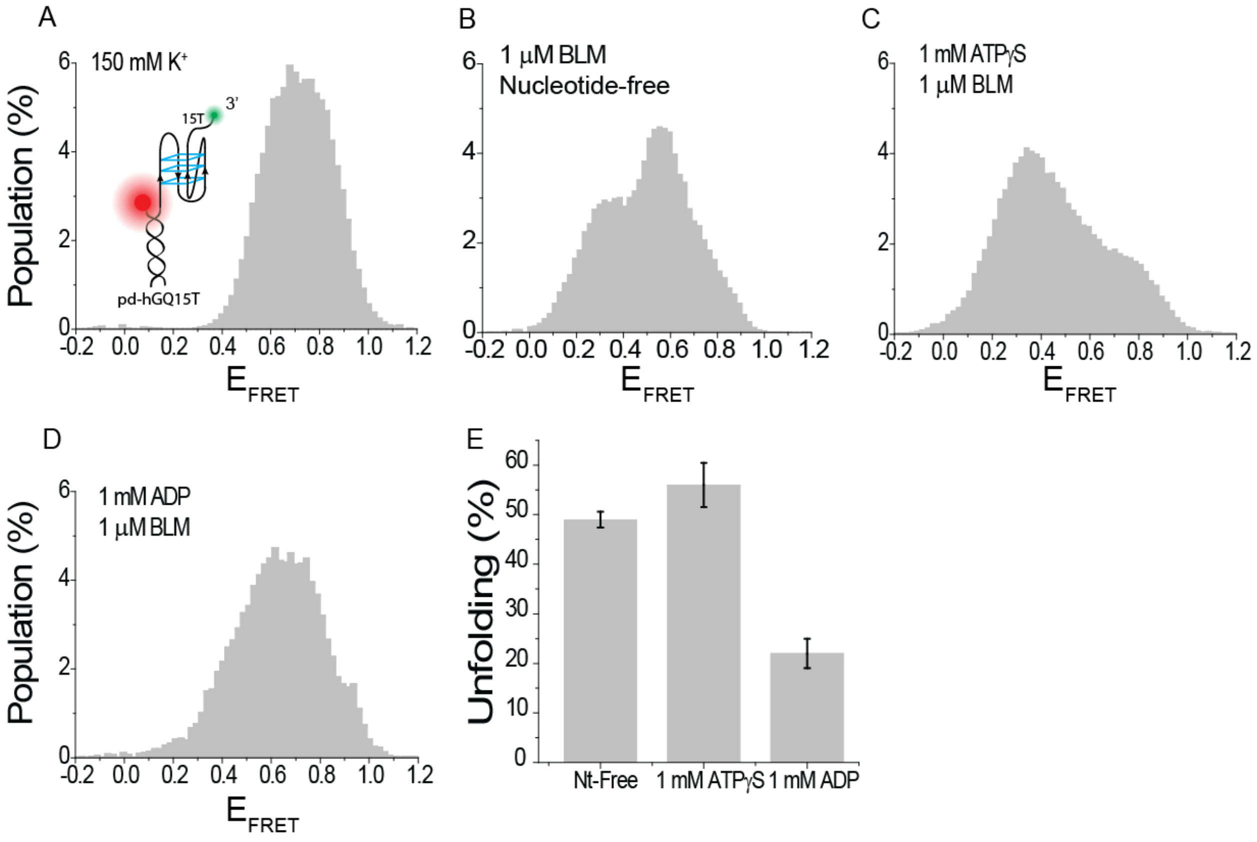

Figure S14. BLM-mediated GQ unfolding on a substrate containing a 15-nt overhang (pd-hGQ15T) at 150 mM K^+. (A) pd-hGQ15T folds into a stable GQ at 150 mM K^+, resulting in a high FRET peak. (B)-(D) smFRET data at 1 μM BLM for different nucleotide states. (B) Nt-free state. (C) 1 mM ATPγS. (D) 1 mM ADP. (E) The folded GQ data shown in (A) is subtracted from respective nucleotide states shown in (B)-(D) to obtain the unfolded populations in these nucleotide states.

FRET Histograms and Subtraction Analysis for BLM and ATPγS Titration in 50 mM K^+ for pd-hGQ12T

Figure S15 shows the histograms for ATPγS and BLM concentrations for pd-hGQ12T construct at 50 mM K^+. Figure S16 shows the corresponding histograms of subtraction analysis which we used to quantify BLM-mediated GQ unfolding. The nucleotide and BLM concentrations used for each histogram are indicated on the graphs.

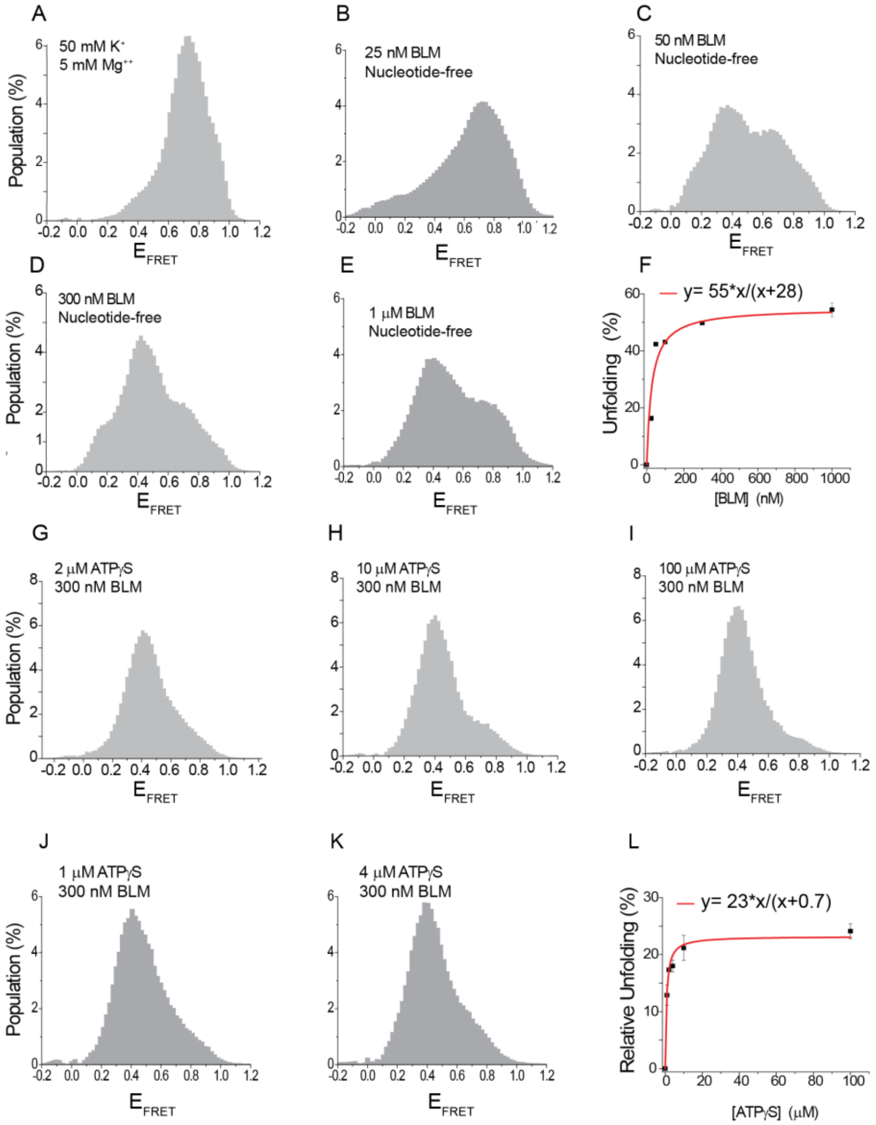

Supplementary Figure S15: smFRET histograms showing the ATPγS and BLM concentrations we studied. All data were taken at 50 mM K^+. **(A)-(E)** Data at the nt-free state where BLM is titrated at 0, 25 nM, 50 nM, 300 nM, and 1 μM concentration, respectively. **(F)** Langmuir binding isotherm fit to the data shown in (A)-(E). **(G)-(K)** Data in the presence of 300 nM BLM where ATPγS is titrated at 1 μM, 2 μM, 4 μM, 10 μM, and 100 μM, respectively. **(L)** Langmuir binding isotherm fit to the data shown in (G)-(K).The nucleotide and BLM concentration used for each histogram is indicated on the graphs.

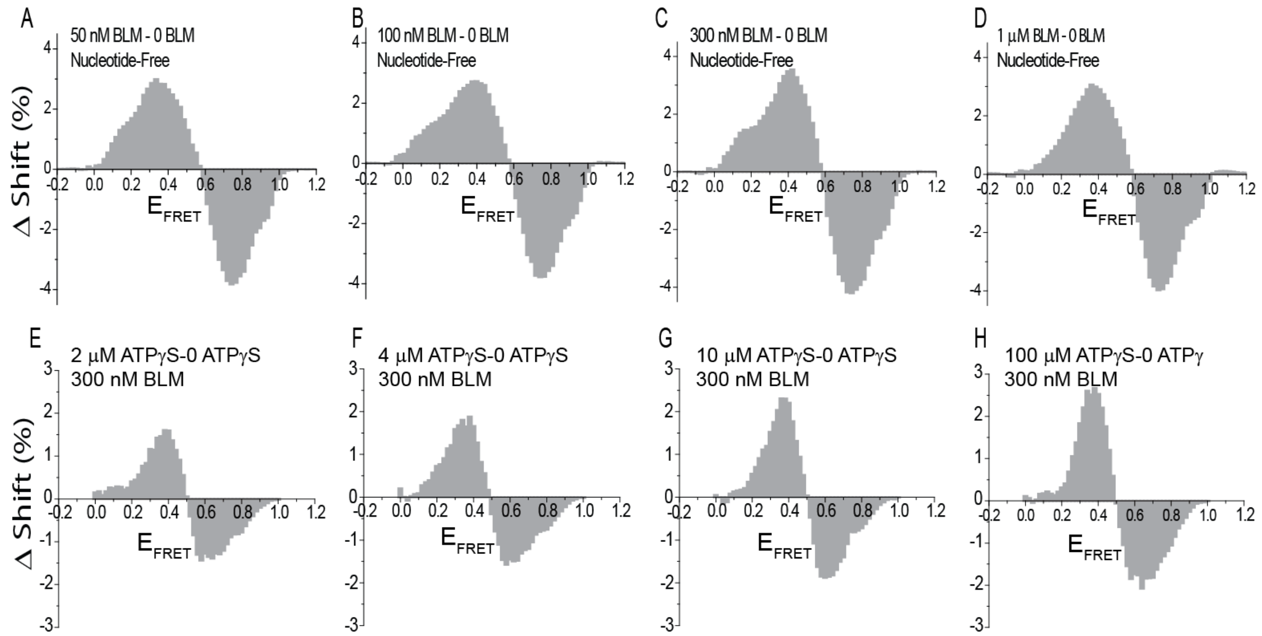

Supplementary Figure S16: Subtraction analysis performed on the data shown in Supplementary Figure S15. Four representative concentrations for BLM or ATPγS titration are shown. **(A)-(D)** Subtraction analysis for data taken at 50 nM, 100 nM, 300 nM, and 1 μM BLM concentration in the nt-free state, respectively. The reference state for this analysis was the nt-free state in the absence of BLM. **(E)-(H)** Subtraction analysis performed for data taken at 2 μM, 4 μM, 10 μM, and 100 μM ATPγS concentration, respectively, in the presence of 300 nM BLM. The nt-free state, e.g. zero ATPγS, in the presence of 300 nM BLM was used as the reference state for the subtraction analysis in (E)-(H). The BLM and ATPγS concentration used for each histogram are indicated on the graphs.

Quantifying the Change in BLM Binding Affinity to ssDNA at 150 mM K^+ *vs*. 50 mM K^+

Reducing the monovalent ion concentration from 150 mM to 50 mM does not only reduce the GQ stability but also increases the binding affinity of BLM to the overhang ssDNA. As both effects would result in increased BLM-mediated GQ unfolding in 50 mM, we sought to estimate the relative contribution of each effect. We used a partial duplex DNA with a 15 nt long unstructured polythymine ssDNA segment for these studies (pd-polyT15), and measured the equilibrium constant of BLM for 50 mM and 150 mM Na^+. Figure S17 shows these data which result in a 4.7 fold increase in K_{eq} in 150 mM Na^+ compared to 50 mM Na^+. In comparison BLM-mediated GQ unfolding increased by 10.8 fold in 50 mM K^+ compared to 150 mM K^+. Therefore, we conclude that the GQ stability contributes approximately a factor of 2.3 in this 10.8 fold increase in K_{eq}.

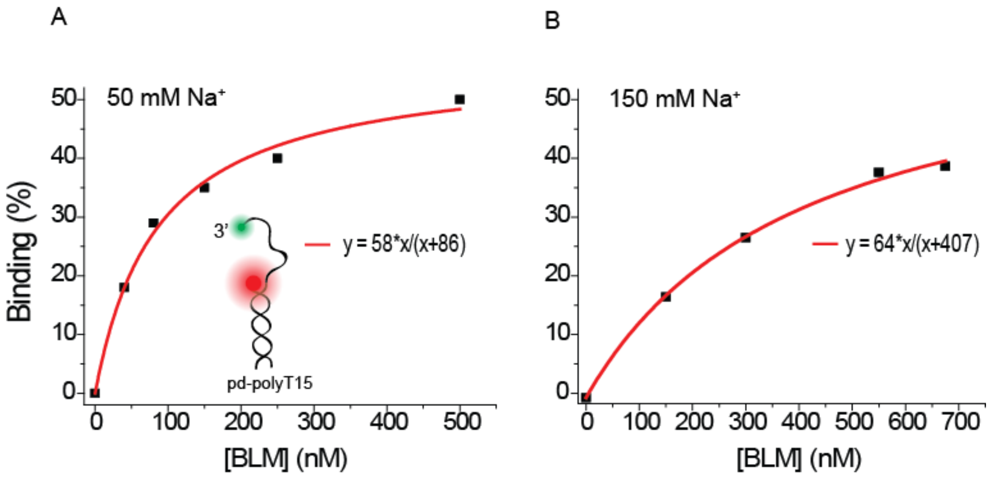

Supplementary Figure S17: The change in BLM binding affinity to ssDNA due to lower ionic strength is characterized by an smFRET assay. BLM binding to pd-polyT15 is quantified in **(A)** 50 mM Na^+ and **(B)** 150 mM Na^+. The fits are Langmuir binding isotherms.

BLM Binding to a Partial Duplex DNA with 5'-end

In order to determine whether the reduced BLM-mediated GQ unfolding observed for the GQ construct with 5' overhang is related to the ability of BLM to bind the 5'-overhang, we performed smFRET measurements similar to those presented in Figure 3 of the manuscript. Figure S18 shows these data and a schematic of the construct used in these studies, pd-polyT15-5', which is a partial duplex DNA with 15 thymine long ssDNA overhang with 5' polarity. pd-polyT15-5' is formed by hybridizing polyT15-5' and DNA-Stem strands given in Table 1. Figure S18-A shows that 1 μM BLM does not bind to pd-polyT15-5' in 150 mM K^+ in the nt-free state. The data representing these conditions are shown with dashed red curve in Figure S18-A as this curve has a nearly perfect overlap with the black solid curve representing the folded state in the absence of BLM. The solid blue curve in Figure S18-A represents the 1 mM ATPγS state which shows a small amount of BLM binding to the overhang. Reducing the K^+ concentration to 50 mM did not have a significant influence on BLM-binding to pd-polyT15-5' in the nt-free state however, significant binding (53%) is observed in the 1 mM ATPγS state (Figure S18-B).

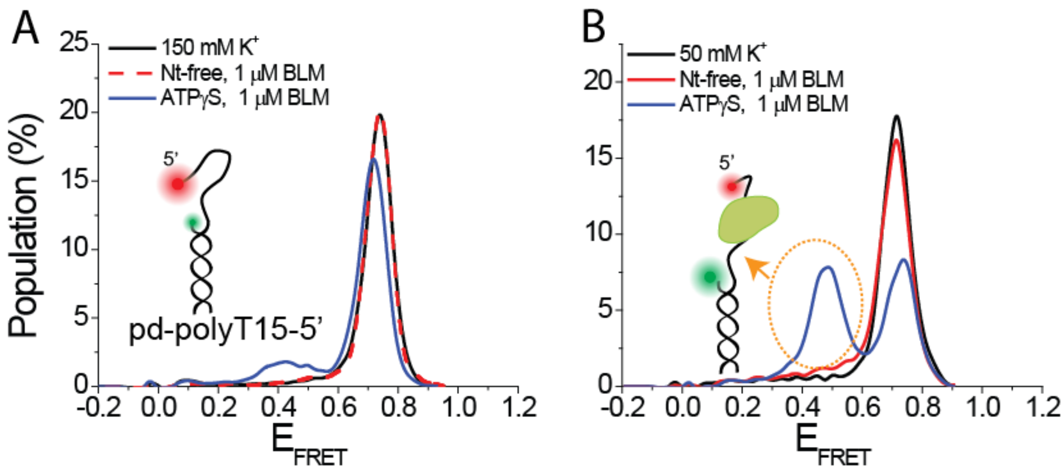

Supplementary Figure S18: BLM binding to pd-polyT15-5' in 150 mM and 50 mM K^+. **(A)** 1 μM BLM does not bind to pd-polyT15-5' in the nt-free state in 150 mM K^+, but a small amount of binding is observed in 1 mM ATPγS. (B) Reducing the ion concentration to 50 mM K^+ results in significant BLM binding in the ATPγS state (blue curve) but not in the nt-free state (red curve).

Interactions of *E. coli* RecQ and human RECQ5 with GQ

Figure 7 of the manuscript shows interactions of *E. coli* RecQ and human RECQ5 with pd-hGQ12T in 50 mM Na^+. These measurements were initially performed with 50 mM K^+ or 150 mM K^+, which resulted in no detectable GQ unfolding. Figure S19 shows these data in 50 mM K^+ for RecQ and 150 mM K^+ for RECQ5 in both nt-free and 1 mM ATPγS states. For both RecQ and RECQ5 cases, the FRET peak representing the folded state shifts towards lower FRET. However, this shift is not large enough to account for GQ unfolding and most likely represents binding of RecQ or RECQ5 to the overhang. As shown in Figure S1, the unfolded state for a DNA of this length in 50 mM K^+ should have $E_{FRET}<0.40$ since the unfolded state at 150 mM K^+ has $E_{FRET}=0.40$. The protein bound state should have $E_{FRET} \approx 0.20$. Since the shift we observe results in $E_{FRET}>0.40$, these shifts do not represent unfolding of the GQ.

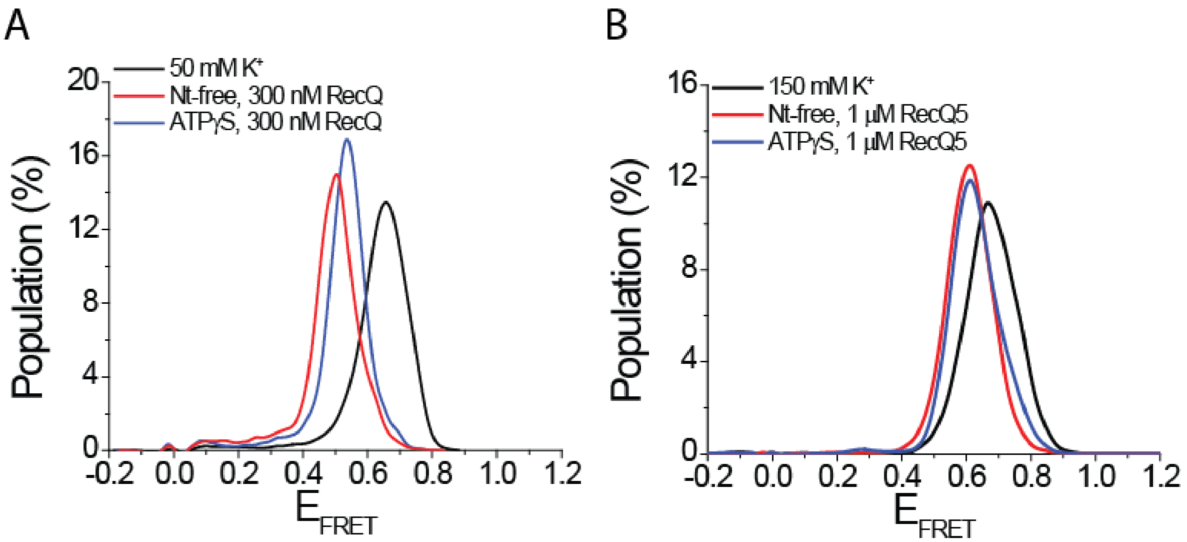

Supplementary Figure S19: smFRET histograms showing interactions of RecQ and RECQ5 with pd-hGQ12T in nt-free and ATPγS states. (A) 300 nM RecQ does not unfold this GQ in 50 mM K^+ in either nt-free or ATPγS state. The shift towards lower FRET upon adding RecQ represents binding of RecQ to the 12 nt overhang. (B) 1 μM RECQ5 does not unfold pd-hGQ12T in either nt-free or ATPγS states. The slight shift towards lower FRET upon adding RECQ5 represents binding of RECQ5 to the 12 nt overhang.

BLM-GQ Complex Remains Dynamic at All BLM Concentrations

smFRET time traces were examined to determine whether at high BLM concentrations, multiple BLM molecules bind to the ssDNA that becomes available upon GQ unfolding. This would essentially result in a permanently unfolded GQ state. However, smFRET time traces show that even at 1 μM BLM the system remains dynamic in saturating nucleotide concentrations. Figure S20 shows three example time traces that demonstrate these dynamics in the nt-free, 100 μM AMP-PNP, or 100 μM ATPγS. These data demonstrate that GQ unfolds and refolds multiple times within 1-2 minute observation time, ruling out the possibility that GQ is locked in a permanently unfolded state.

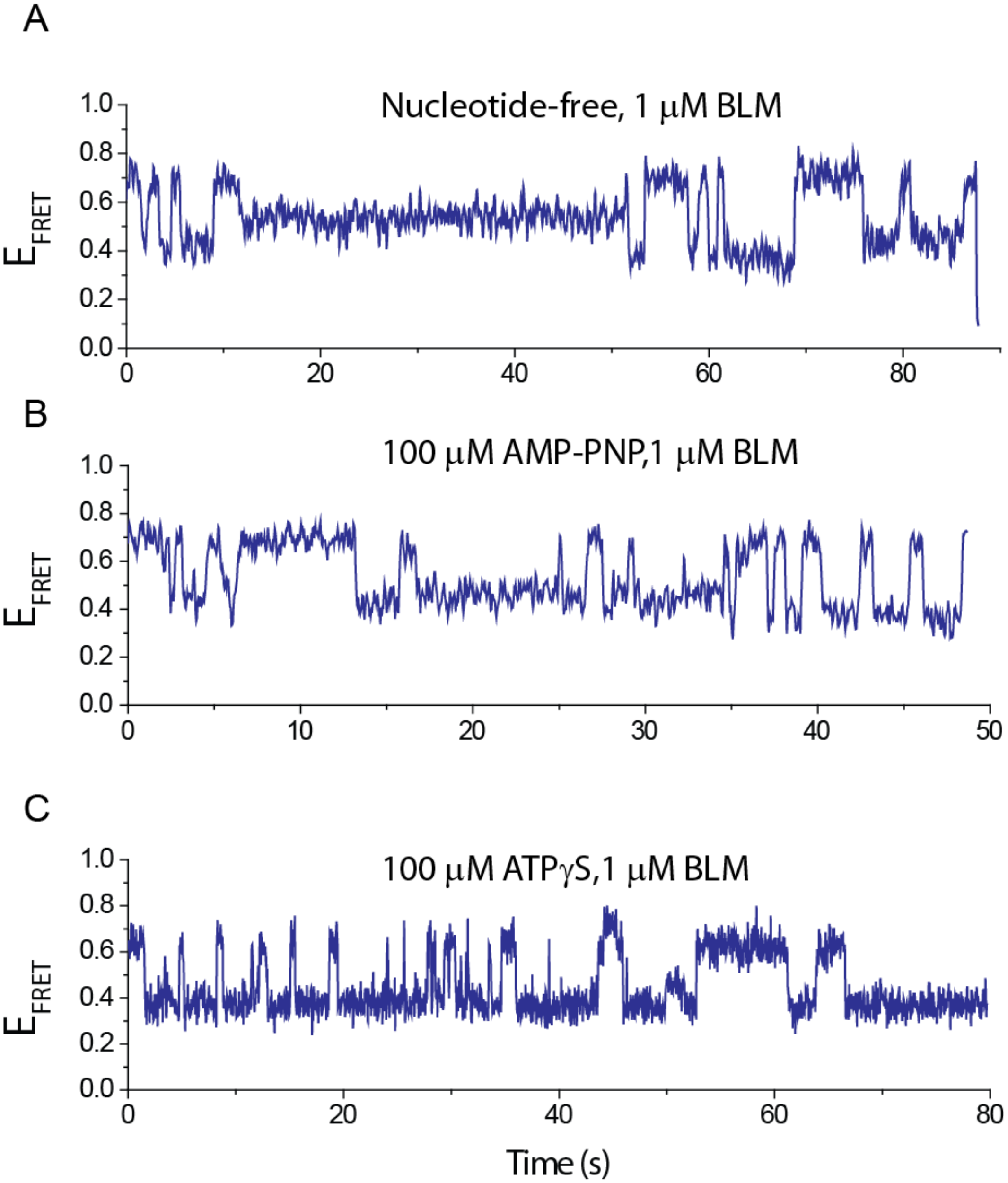

Supplementary Figure S20: Example smFRET time traces in 1 μM BLM in **(A)** Nt-free; **(B)** 100 μM AMP-PNP; and **(C)** 100 μM ATPγS. The dynamics observed in such traces demonstrate that GQ is not permanently unfolded due to binding of multiple BLM molecules.
